# Instrumented assessment of motor function in dyskinetic cerebral palsy: a systematic review

**DOI:** 10.1186/s12984-020-00658-6

**Published:** 2020-03-05

**Authors:** Helga Haberfehlner, Marije Goudriaan, Laura A. Bonouvrié, Elise P. Jansma, Jaap Harlaar, R. Jeroen Vermeulen, Marjolein M. van der Krogt, Annemieke I. Buizer

**Affiliations:** 1 Department of Rehabilitation Medicine, Amsterdam Movement Sciences, Amsterdam UMC, PO Box 7057, Amsterdam, 1007MB The Netherlands; 2 Department of Human Movement Sciences, Faculty of Behavioural and Movement Sciences, Vrije Universiteit Amsterdam, Amsterdam Movement Sciences, Amsterdam, The Netherlands; 3 Medical Library, Vrije Universiteit Amsterdam, Amsterdam, The Netherlands; 4 Department of Epidemiology and Biostatistics, Amsterdam Public Health research institute, Amsterdam UMC, Location VUmc, Amsterdam, The Netherlands; 5 Department of Biomechanical Engineering, Delft University of Technology, Delft, The Netherlands; 6 Department of Neurology, Section of Pediatric Neurology, Maastricht UMC+, Maastricht, The Netherlands

**Keywords:** Dystonia, Choreoathetosis, Technology, Reliability, Validity, Responsiveness, Quantitative assessment, Devices

## Abstract

**Background:**

In this systematic review we investigate which instrumented measurements are available to assess motor impairments, related activity limitations and participation restrictions in children and young adults with dyskinetic cerebral palsy. We aim to classify these instrumented measurements using the categories of the international classification of functioning, disability and health for children and youth (ICF-CY) and provide an overview of the outcome parameters.

**Methods:**

A systematic literature search was performed in November 2019. We electronically searched Pubmed, Embase and Scopus databases. Search blocks included (a) cerebral palsy, (b) athetosis, dystonia and/or dyskinesia, (c) age 2–24 years and (d) instrumented measurements (using keywords such as biomechanics, sensors, smartphone, and robot).

**Results:**

Our search yielded 4537 articles. After inspection of titles and abstracts, a full text of 245 of those articles were included and assessed for further eligibility. A total of 49 articles met our inclusion criteria. A broad spectrum of instruments and technologies are used to assess motor function in dyskinetic cerebral palsy, with the majority using 3D motion capture and surface electromyography. Only for a small number of instruments methodological quality was assessed, with only one study showing an adequate assessment of test-retest reliability. The majority of studies was at ICF-CY function and structure level and assessed control of voluntary movement (29 of 49) mainly in the upper extremity, followed by assessment of involuntary movements (15 of 49), muscle tone/motor reflex (6 of 49), gait pattern (5 of 49) and muscle power (2 of 49). At ICF-CY level of activities and participation hand and arm use (9 of 49), fine hand use (5 of 49), lifting and carrying objects (3 of 49), maintaining a body position (2 of 49), walking (1 of 49) and moving around using equipment (1 of 49) was assessed. Only a few methods are potentially suitable outside the clinical environment (e.g. inertial sensors, accelerometers).

**Conclusion:**

Although the current review shows the potential of several instrumented methods to be used as objective outcome measures in dyskinetic cerebral palsy, their methodological quality is still unknown. Future development should focus on evaluating clinimetrics, including validating against clinical meaningfulness. New technological developments should aim for measurements that can be applied outside the laboratory.

## Background

Cerebral palsy (CP) is the most common physically disabling condition in childhood, with a prevalence of approximately two in every 1000 live births in Europe [1]. Dyskinetic CP accounts for 6–15% of all children with CP and is the second most common form after spastic CP (85%) [1–3]. A majority of children with dyskinetic CP have a lesion in the basal ganglia and thalamus or both [4]. Children and young adults with dyskinetic CP experience limitations in mobility and manual ability due to motor impairments. These motor impairments are characterized by involuntary movements and changes in muscle tone (i.e. dystonia and choreoathetosis) [2, 5]. The severity of motor impairments and limitations in mobility and manual ability is wide-ranging in dyskinetic CP. However, the majority of children with dyskinetic CP are severely affected, with about 70–80% classified within the Gross Motor Functioning Classification System (GMFCS) [6] level IV-V (i.e. non-ambulatory) and the Manual Ability Classification System (MACS) [7] level IV-V (i.e. severely impaired manual ability) [8, 9].

Several scales are currently used to describe the severity of dystonia in dyskinetic CP [10]. Examples are the Barry-Albright Dystonia Scale (BADS) [11], Dyskinesia Impairment Scale (DIS) [12], Burke-Fahn-Marsden Dystonia Rating Scale (BFMDRS) [13], Hypertonia Assessment Tool (HAT) [14, 15], and Unified Dystonia Rating Scale (UDRS) [16]. The DIS in addition to dystonia also assesses choreoathetosis [12]. Clinical scales are often combined with questionnaires such as the Pediatric Evaluation of Disability Inventory (PEDI) [17] and Child Health Index of Life with Disabilities (CPCHILD) [18], and performance based outcome measures such as Quality of Upper Extremity Skills Test (QUEST) [19]. Also individualized outcome measures (Goal attainment scaling (GAS) [20] or Canadian Occupational Performance Measure (COPM) [21] are used to evaluate treatment outcome in dyskinetic CP [22–26].

The current clinical scales (e.g. BADS, DIS and BFMDRS) are based on the observation by a clinician [10]. Although treatment (e.g. intrathecal baclofen and deep brain stimulation) targets a decrease of dystonia and choreoathetosis the effects of treatment have mainly been found on individualized outcome measures (e.g. GAS and COPM) and less or not at all on clinical scales [27, 28]. Outcomes of the clinical scales measuring dystonia and choreoathetosis are subjective, i.e. dependent on the personal judgement and experience of the rater. Therefore, it might be useful to measure motor impairments in this patient group using objective measurements.

Another reason for the discrepancy in outcome between clinical scales and individualized outcome measures might be that the severity of abnormal movements varies over time and is exacerbated by external stimuli, such as stress, pain and noise [4], therefore improvement in dystonia and choreoathetosis might be difficult to capture at one time point in the clinical environment. Ideally, measuring at home might result in more meaningful and precise outcome.

Kinematic measures within a laboratory environment (i.e. gait and upper limb functional analysis) are frequently performed within a general population of CP and seem to become more common in dyskinetic CP as well [29]. Devices such as surface electromyography (sEMG) and wearable sensors may offer additional opportunities to objectively quantity dyskinetic movements (i.e. as functions of the musculoskeletal system) such as dystonia and choreoathetosis in dyskinetic CP. Wearable sensors may also allow for assessment of activities and participation outside the laboratory environment.

Although these techniques are promising, there is currently no consensus which outcome parameters are relevant in the assessment of dyskinetic movements or related activity limitations and participation restrictions in dyskinetic CP. As a first step we deem it necessary to inventory the outcome parameters that are currently used in studies using instrumented measures of motor function. To describe functioning in dyskinetic cerebral palsy, the ICF (international classification of functioning) provides a useful framework [30]. It can be used to classify assessment tools as to which aspect of functioning they measure [30]. The ICF is a classification system of functioning and disability, distinguishing between (a) body function and structure, (b) activities and participation, and (c) components of contextual factors i.e. environmental factors and personal factors [31]. We considered the following chapters of the ICF to be relevant for the assessment of motor function in dyskinetic CP: (a) within body function and structure: Neuromusculoskeletal and movement-related functions, focusing on muscle and movement functions and (b) within activities and participation: Mobility, especially changing and maintaining body position, carrying, moving and handling objects and walking and moving. The ICF children and youth version (ICF-CY) has derived from the ICF expanding the coverage of the main ICF volume by providing specific content and additional detail to more fully cover the body functions and structures, activities and participation, and environments of particular relevance to infants, toddlers, children and adolescents [31]. We choose to use the ICF-CY categories to cover the age range from preschool children up to young adults. See Table 1 for codes and definitions of ICF-CY [31].

**Table 1:** Considered relevant categories of the international classification of functioning, disability and health for children and youth (ICF-CY) [31] for dyskinetic cerebral palsy related to movement disorders

Body function and structure							
*Neuromusculoskeletal and movement-related functions (Chapter 7)*							
Muscle functions (b730-b749)				Movement functions (b750-b789)			
b730	b735	b740	b750	b755	b760	b765	b770
Muscle power^a^	Muscle tone	Muscle endurance	Motor reflex	Involuntary movement reaction	Control of voluntary movement	Involuntary movement	Gait pattern
Function related to the force generated by the contraction of a muscle or muscle group^b^.	Functions related to the tension present in the resting muscles and the resistance offered when trying to move the muscles passively^c^.	Functions related to sustaining muscle contraction for the required period of time^d^.	Functions of involuntary contraction of muscles automatic-ally induced by specific stimuli^e^.	Functions of involuntary contractions of large muscles or the whole body induced by body position, balance and threatening stimuli^f^.	Functions associated with control over and coordination of voluntary movements^g^.	Functions of unintentional, non- or semi-purposive involuntary contractions of a muscle or group of muscles^h^.	Functions of movement patterns associated with walking, running or other whole body movements^i^.
Activities and participation							
*Mobility (Chapter 4)*							
Changing and maintaining body position (d410-d429)		Carrying, moving and handling objects (d430-d449)			Walking and moving (d450-d469)		
d410	d415	d430	d440	d445	d450	d455	d465
Changing basic body position	Maintaining a body position	Lifting and carrying objects	Fine hand use	Hand and arm use	Walking	Moving around	Moving around using equipment
Getting into and out of a body position and moving from one location to another.	Staying in the same body position as required.	Raising up an object or taking something from one place to another.	Performing the coordinated actions of handling objects, picking up, manipulating and releasing them using one’s hand, fingers and thumb.	Performing the coordinated actions required to move objects or to manipulate them by using hands and arms.	Moving along a surface on foot, step by step, so that one foot is always on the ground.	Moving the whole body from one place to another by means other than walking.	Moving the whole body from place to place, on any surface or space, by using specific devices designed to facilitate moving or create other ways of moving around.

^a^Note that power in physics is defined as energy output per unit of time, or the rate of doing work. Strength (force or torque output) and power (work/time) are separate physical parameters. However, the ICF-CY does not distinguish between strength and power. Therefore articles measuring strength were included in the category muscle power

Inclusion:

^b^Functions associated with the power of specific muscles and muscle groups, muscles of one limb, one side of the body, the lower half of the body, all limbs, the trunk and the body as a whole

^c^Functions associated with the tension of isolated muscles and muscle groups, muscles of one limb, one side of the body and the lower half of the body, muscles of all limbs, muscles of the trunk, and all muscles of the body; impairments such as hypotonia, hypertonia and muscle spasticity

^d^Functions associated with sustaining muscle contraction for isolated muscles and muscle groups, and all muscles of the body

^e^Functions of stretch motor reflex, automatic local joint reflex, reflexes generated by noxious stimuli and other exteroceptive stimuli; withdrawal reflex, biceps reflex, radius reflex, quadriceps reflex, patellar reflex, ankle reflex, appearance and persistence of reflexes

^f^Functions of postural reactions, righting reactions, body adjustment reactions, balance reactions, supporting reactions, defensive reactions

^g^Functions of control of simple voluntary movements and of complex voluntary movements, coordination of voluntary movements, supportive functions of arm or leg, right left motor coordination, eye hand coordination, eye foot coordination; impairments such as control and coordination problems

^h^Functions of involuntary contractions of muscles; impairments such as tremors, tics, mannerisms, stereotypies, motor perseveration, chorea, athetosis, vocal tics, dystonic movements and dyskinesia

^i^Walking patterns and running patterns

### Aim of review

The objective of this review is to investigate which instrumented measurements are available at all levels of the ICF-CY to assess motor function in children and young adults with dyskinetic CP. Additionally, we aim to provide an overview of the parameters that can be extracted from these instrumented measurements.

## Methods

### Search and selection

A literature search was performed in November 2019. We electronically searched: Pubmed, Embase and Scopus. The search strategy for Pubmed has been published along with the study protocol [32]. For the other databases the same search strategy was used, but modified to the corresponding database. The searches are provided in the supplementary materials (Additional file 1). In brief, the search blocks included (a) diagnosis (i.e. cerebral palsy), (b) movement disorder (i.e. dyskinesia, athetosis and dystonia), (c) age (i.e. 2–24 years) and (d) instrumented measurements with keywords such as biomechanics, accelerometer, velocity, speed, electromyography, sensors, smart phone, computer, and robot. To also cover literature where cerebral palsy was not mentioned in the title or abstract, population was defined by: (a) and (b) or (b) and (c).

The search results were imported into Endnote X8 (Clarivate Analytics, Boston, USA). After removal of duplicates, all titles and abstracts were transferred to Rayyan (Qatar Computing Research Institute, Qatar), a free web application for systematic reviews [33]. In Rayyan two reviewers (HH, MG) independently screened titles and abstracts against the inclusion criteria. The inclusion criteria are presented in Table 2. The studies that were selected by HH and MG were retrieved in full text and their citation information imported in Endnote as a second database. The reference lists of all studies retrieved in full text as well relevant secondary research (i.e. reviews) were screened for additional studies. The full text of selected citations were then assessed in detail against the same inclusion criteria defined in the PICOS (Participants, Intervention, Comparison, Outcome, and Study design) framework [34] (Table 2) by both reviewers (HH, MG). Any disagreements that occurred between these reviewers at each stage of the study selection process was resolved through discussion with a third and if necessary fourth reviewer (LB, AB).

**Table 2:** Inclusion and exclusion criteria defined in the PICOS (Participants, Intervention, Comparison, Outcome, Study design) framework

	Description	Inclusion/Exclusion criteria
Participants	Dyskinetic CP, 2–24 years	-The study sample or an substantial number of subjects (minimal 50%) are represented in the study population or in a sub-study population that is separately analyzed; -As definition of dyskinetic CP is not always clear also studies describing dystonia due to CP are included
Intervention	Instrumented measurements to assess movement function and related activities/participation	-Imaging techniques (e.g. MRI) were excluded -Studies that only use video recording without computerized analyzing techniques but purely to score from the video through observation were excluded.
Comparison	No control group or comparison is required	-Comparison to a clinical test, a control group or the effect of intervention assessed by the methods will be reported but if there is none, the method is still listed in the review
Outcome	Outcomes measured in one of the ICF-CY level reported in Table 1 (i.e. muscle or movement function, changing and maintaining body position, carrying, moving and handling objects, (fine) hand and arm use or walking and moving with or without equipment)	-Other categories of ICF-CY (e.g. mental functions, sensory functions and pain, speech, communication or self-care) were excluded
Study design	Original research studies are included, peer reviewed full text and conference abstracts with sufficient information on used methodology and participants	-No restrictions on the type of studies, including technical reports, case studies, case-control studies and intervention studies -If both an abstract and full text article were published on the same data/methodology only the full text article was selected -Articles published in languages other than English were excluded

*CP* cerebral palsy, *MRI* magnetic resonance imaging, *ICF-CY* international classification of functioning for children and youth

### Data extraction and assessment of methodological quality

We extracted relevant information from each included paper in a custom-made Excel based (Microsoft Office, Microsoft, Redmond, WA, USA) data extraction form. Information regarding patient characteristics, assessed ICF-CY categories (Table 1), body region, outcome parameters, used instruments/technologies/software, and primary aim of the study was extracted. Studies may be categorized in more than one ICF-CY category when multiple experiments are performed or an experiment includes outcome parameters in different categories. Measurement properties of the available techniques (i.e. validity, reliability, responsiveness and measurement error) were assessed with the COnsensus-based Standards for the selection of health Measurement INstruments (COSMIN) checklist of bias [35]. Data extraction was done by one reviewer (HH) and audited by a second reviewer (MG).

## Results

**Fig. 1:**
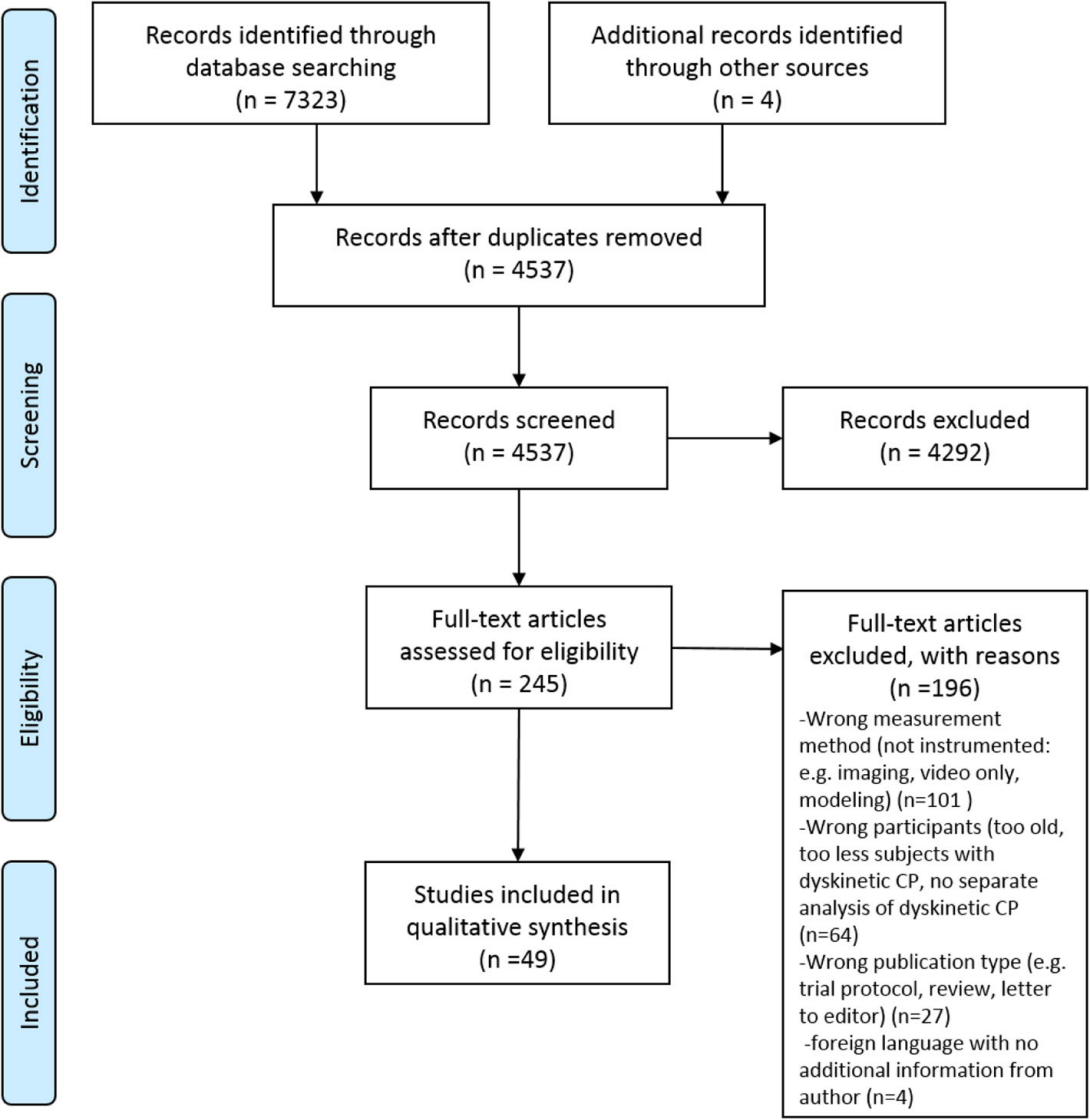
PRISMA flow diagram for information through the different phases of study selection

An overview of the search and selection process is shown in Fig. 1 using a Preferred Reporting Items for Systematic Reviews and Meta-analyses (PRISMA) flow diagram [34].

**Fig. 2:**
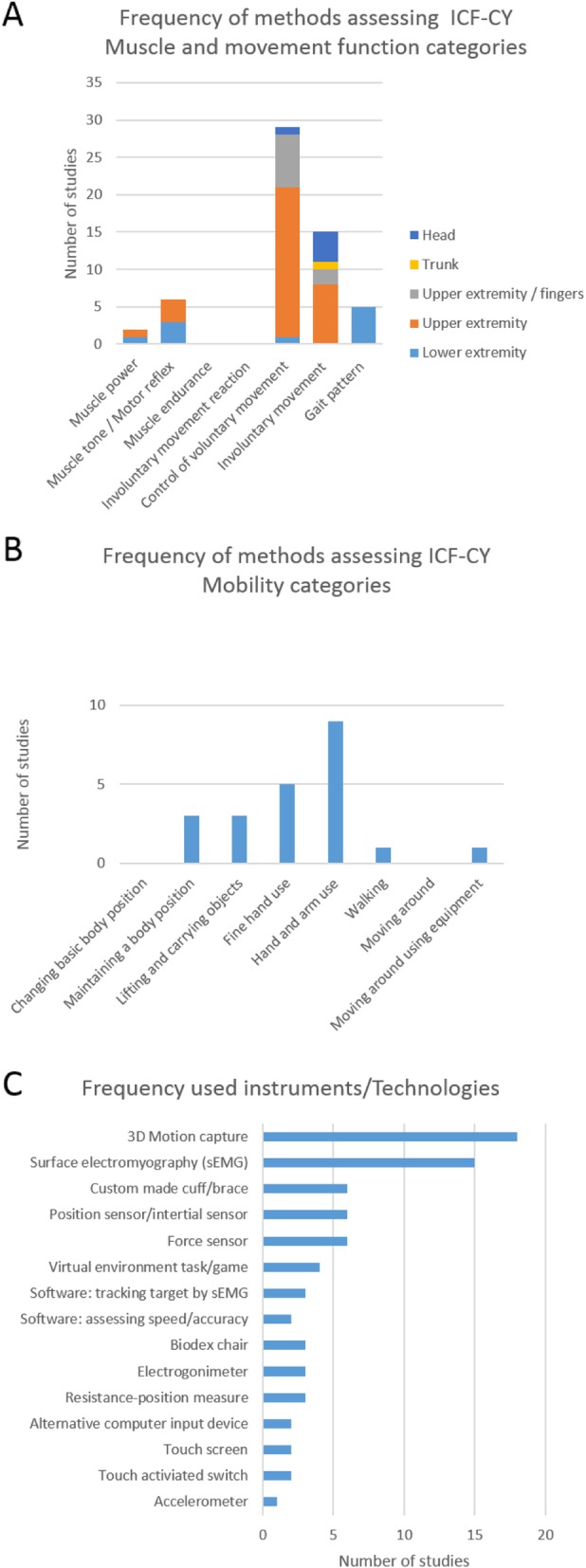
Frequency of instrumented assessed categories within the international classification of functioning, disability and health for children and youth (ICF-CY) for dyskinetic cerebral palsy **a**: Muscle and movement functions categories, **b**: Mobility categories and **c**: Frequency of used instruments and technologies

After removal of duplicates, our search yielded 4537 records, of which a total of 245 were included in full-text for further eligibility assessment. Subsequently, 196 articles were excluded based on the following main reasons: 1) no instrumented measurements were used to assess movement function, 2) the participants were too old, 3) too many participants did not have the diagnosis of dyskinetic CP, or 4) in case of mixed group of participants with CP, no sub analysis of dyskinetic group was performed. Finally, 49 articles [36–84] were included in the review. A summary of the included studies is provided as supplementary material (Additional file 2). Overall the sample size of the included studies was low with majority of studies including 10–20 participants. Figure 2 shows an overview of frequency of the ICF-CY categories and frequency of used instruments and technologies.

### Body function and structures

#### Muscle functions

##### Muscle power

Torques and force levels were assessed by force sensors in the lower [38] and upper extremity [45]. In the lower extremity, maximum isometric flexion and extension torques of the knee [38] were analyzed. In the upper extremity, force variability as well as change of force variability with increased force levels of the biceps were studied [45].

From a biomechanical point of view strength (force or torque output) and power (work/time) are separate physical parameters. However, the ICF-CY does not distinguish between strength and power. Therefore articles measuring torque or force were included in the category muscle power.

##### Muscle tone / motor reflex

Muscle tone and motor reflex were measured in both upper and lower extremity, more specifically at elbow [44, 46, 51], knee [36–38] and ankle [37]. The most frequently used method was sEMG alone or combined with force sensors or position measures [36–38, 44, 46, 51]. These measurements intended to distinguish between spastic and dyskinetic CP [36–38, 51], to determine the relation of muscle tone and motor reflex in dyskinetic CP [46], and/or the influence of muscle tone and motor reflex on control of voluntary movement [44, 51].

#### Movement functions

##### Involuntary movement

Involuntary movement was mainly quantified by determining the overflow of muscle activation that is not directly required for a task, thus resulting in involuntary movement. This was mostly measured as kinematic overflow by 3D motion analysis [51, 54, 60, 61] and overflow of muscle activation by sEMG [44, 71–74]. Different stimuli were used to trigger overflow e.g. movement of contralateral arm, hand, fingers, antagonist muscles [44, 51, 54, 60, 61, 71, 73, 74], and eye-blinking [61]. Other studies measured the involuntary movement component during voluntary movements [48, 82], while holding the arm in a raised position and during lying in rest [62]. In addition, movement parameters were measured during quiet sitting using perturbations as a sudden noise [80], closing the eyes [83] or computer use [84].

##### Control of voluntary movement

Control of voluntary movement was measured for the upper extremity [43, 44, 47–60, 63–67, 78, 79]*,* for the fingers [69–77]*,* for the lower extremity [43], head [81] and trunk [78, 79] in a variety of ways:

(a) Position and joint angle measurements were performed using 3D motion tracking and other measures like electrogoniometry and shape tape to assess spatiotemporal and kinematic parameters during different upper extremity tasks [48, 51–56, 58–60, 63, 75, 76, 78, 79] (Additional file 7). Thorax position and stability of the trunk during reaching was assessed in two studies [78, 79] (Additional file 7).

(b) Virtual reality games and touch screen tracking games were also used to evaluate voluntary movement function of upper extremity, finger, and head movements. Several input and output devices were used i.e. a manipulandum controlled by arm movement [49, 50], a touch screen tablet operated by the index finger [69, 70], a virtual handwriting system [77], touch activated switches [65–67] and different kind of mice [81]. These devices were used to assess a range of outcome parameters mainly studying movement time and/or accuracy (Additional file 7).

(c) Muscle activity was measured in several studies by sEMG to assess the contribution of muscle activity to task performance. This was done during elbow extension movements [44], during activating a switch by touch [65], during a finger-to-nose task [59] and during a 8-figure writing task [75, 76].

(d) sEMG was also used to assess voluntary muscle activation to control tracking games on a (computer) screen e.g. tracking a target by activating biceps and triceps [47], keeping an indicator in a central position by biceps activation [43], control of position and movement of a line by left and right biceps activation [64] or tracking a target by isometrically activating intrinsic muscles [71–74].

##### Gait pattern

Parameters to assess gait pattern were all measured by marker based 3D motion capture systems. The marker models used and parameters derived from it differed between studies, but consisted mainly of spatiotemporal and kinematic parameters [38–42] (Additional file 7).

### Activities and participation

Control of voluntary and involuntary movement was assessed within functional activities in some studies and these studies were classified within the following categories of activities and participation with the ICF-CY: (a) maintaining a body position [79, 80, 83], (b) lifting and carrying objects [52, 53, 55, 56], (c) fine hand use [69, 70, 75–77]; (d) hand and arm use [51, 54, 57–60, 63, 65–67, 78, 79]. One study measured not only gait pattern but also walking velocity and was therefore classified within the ICF-CY category (e) walking [38]. (f) Moving around using equipment (i.e. electric wheelchair) was assessed in one recent study [68]. No studies were found that assessed ICF-CY categories of changing basic body position and moving around.

### Assessment of methodological quality

Initially we aimed to assess methodological quality using the COSMIN checklist [35] However, most papers did not have as primary aim to determine measurement properties and provided little to no information for a formal scoring of the measurement properties of described instrumented measurements. We therefore decided to give an indication concerning construct validity by (a) describing which parameters were compared to a clinical instrument (i.e. hypotheses testing, convergent validity) and (b) describing which parameters were used to compare a dyskinetic CP group to a control group or to distinguish between subgroups (e.g. between spastic and dyskinetic CP, i.e. hypotheses testing, discriminative validity) (Additional file 7). We also extracted information on whether test-retest or intra-session reliability was assessed and/or if information on the measurement error was available (Additional file 7). An indication of responsiveness of the used instrumented methods is given by describing if the method has been used in evaluation of an intervention (Additional file 7).

The results of studies that report the correlation of measured parameters to a clinical comparator instrument (e.g. BADS, UDRS, BFMDRS, MACS) [39, 47, 51–53, 55, 56, 58–62, 69, 71–73] are summarized in Additional file 3. An overview of studies that report on comparison to a control group [37, 39, 40, 44, 45, 47–56, 58–61, 64, 67, 69–71] or distinguish between subgroups within the cerebral palsy (i.e. dyskinetic versus spastic) [38–40, 42, 51, 53, 55, 57, 66, 83] are provided in Additional file 4. In four studies a reliability assessment was performed, either intra-session [53, 66] and /or test-retest reliability [61, 62, 66] (Additional file 5). Only one article used adequate statistical testing to determine reliability of the assessment, i.e. reporting intraclass correlation coefficients (ICC) and a measurement error [61]. Limitations of the remaining studies were that correlation coefficients were provided without testing for a systematic change between sessions [53], the number of subjects used for the reliability analysis were extremely low (*n* = 3) [62, 66], or analyses were performed for the entire group of participants with CP, without making a distinction for dyskinetic CP [53]. Additional file 6 provides an overview of studies used an instrumented method assessing the effects of an intervention (e.g. physical exercise [43], biofeedback [47, 64, 71, 76] splints [57], deep brain stimulation [62], botulinum Toxin Type B [63], different seating types [66, 80] and transcranial direct current stimulation [72–74].

## Discussion

The current review provides an overview of available instrumented measurements to assess motor function in dyskinetic CP, at all levels of the ICF-CY. It can serve researchers and clinicians to make an informed decision about instrumented measurements in dyskinetic CP for their specific purpose. There is a range of instrumented methods to assess motor function in dyskinetic CP, especially for the upper extremity. Most methods assess voluntary movement expressed as spatiotemporal and kinematic parameters and involuntary movements expressed as overflow of muscle activation that is not directly required for a task.

### Muscle functions (muscle power, muscle tone / motor reflex, muscle endurance)

Concerning muscle function, the majority of articles focused on muscle tone and motor reflex. Force and/or sEMG was measured during rest and movement (passive and active; slow and fast velocities) [36–38, 44, 46, 51]. Torques, force levels and force variability were described in two articles and classified within the ICF-CY level of functioning of muscle power [38, 45]. No instrumented method is available to assess muscle endurance in dyskinetic CP. This is surprising since force generation and sustaining muscle contraction for a certain amount of time have an effect on task performance. It would be especially useful to assess muscle strength and power in children with dyskinetic CP, because they present with lower maximal isometric flexion and extension torques in the lower limbs when compared to those with spasticity and typically developing children [38]. In addition to a severely impaired strength, children with dyskinetic CP showed increased force variability [45]. Thus strength measurements could provide important information concerning muscle function in dyskinetic CP.

### Movement functions (control of voluntary movement, involuntary movement, gait pattern)

On the ICF level of functioning, control of voluntary movement was frequently assessed by analyzing muscle activity during different tasks via sEMG analyses [44, 59, 65, 75, 76]. The average of co-contraction [59] and the relative contribution of muscle activity (task correlation index) [75, 76] during a repetitive task are possibly interesting variables to assess in children and young adults with dyskinetic CP. Yet experiments were performed in a small group and further development of methods would be necessary. Other ways to study control of voluntary movement were virtual reality or tracking games with various types of input systems (e.g. sEMG controlled input, a touch screen tablet or head mice). Assessment of tracking error, timing error, movement time, or speed-accuracy using Fitt’s law were frequently employed [47, 49, 50, 64–67, 69–71, 73, 74, 77]. These studies point out that available software (e.g. FittsLawSoftware [85]), assessing point-and-click or drag and drop using the individual computer input device of a participant, could be an interesting option for assessment of control of voluntary movement as part of human-computer interaction in dyskinetic CP.

Gait analysis and upper limb measurements using 3D motion capture systems and sEMG are frequently performed in the general population of CP and several recommendations and protocols are available for measurements of kinematics, including Vicon clinical manager or Plug in gait full body model (Vicon UK), American Society of Biomechanics recommendations for upper extremity motion analysis [86], University of Western Australia’s (UWA) upper limb model [87], upper limb model proposed by Rab et al. [88], upper limb three-dimensional movement analysis (UL-3DMA) [89], ELEPAP clinical protocol [90–92] and Reach & Grasp Cycle [93]. These recommendations and protocols were (partly) used in several of the reviewed articles [38–41, 53, 55–57, 60, 61]. Parameters that were mostly used to assess dyskinetic movements during gait and upper extremity tasks included variability and timing of movement trajectories, jerk, kinematic overflow and overflow measured by sEMG [39, 42, 44, 53, 54, 57, 58, 60, 61, 71, 73, 74]. Some of these parameters have already been shown to have a strong correlation to clinical comparator instruments (r > 0.70) (e.g. variability of ankle trajectory during swing [39], kinematic overflow [51, 60, 61] number of movement units (i.e. acceleration-deacceleration) [52] and movement, reach or hold time [52, 55] (see Additional file 3) and might be interesting for assessment of treatment effects. For that, the reliability including measurement error is important to know, especially the test-retest reliability over different days. An insufficient reliability of assessment instruments can hamper results of clinical trials aiming to assess the efficacy of (new) treatments, if changes due to the intervention are not detected. Two studies assessed reliability of outcome variables but the reliability measurements were performed on the same day [53, 61]. Of these two studies, only one used adequate statistical testing following the COSMIN checklist of bias [61]. We expect that the variability of dyskinetic movements negatively affects test-retest reliability on different days, resulting in a higher measurement error in gait analysis and upper limb measurements for dyskinetic CP compared to the general population of CP. To overcome this limitation measuring for a longer period of time might be necessary in dyskinetic CP.

### Assessment of motor function in severely impaired children

The majority of the reviewed methods have in common that they assessed muscle function and movement during tasks that require some level of understanding of a task instruction, manual ability and/or ambulation. However, it is known that in dyskinetic CP a high percentage of children has severe intellectual disability (about 70%), severe visual impairments (about 40%), and 70–80% are non-ambulatory (GMFCS IV-V) and have a severely impaired manual ability (MACS IV-V) [8, 9]. Therefore for a large group of children and young adults with dyskinetic CP, only few instrumented methods are available. No task performance was required for assessing motor activity during rest with an accelerometer attached to the wrist [62] and for evaluation of seating using 3D motion capture and a pressure measurement system of the back, using an external perturbation by sudden noise to trigger dystonic movements [80]. Other possible options for the more severely impaired group are: assessment of head movement during computer use with a computer interface controlled by head movement (e.g. camera mouse, inertial sensor) [81, 82] or finger movement (e.g. wearable switch) [84] and assessment of movement and muscle function during very simple tasks i.e. operate a touch activated switch, perform an outward-reaching, a finger-to nose task, finger-tapping or eye blinking [59, 61, 63, 65–67, 79]. In summary, instrumented assessment for the severely impaired children and young adults are highly needed.

### Implications and future directions

Using instrumented methods with a hypothesis about pathophysiological aspects in mind may lead to more understanding of the mechanisms behind current treatment and may possibly lead to new treatments or improvement of current treatments. It has been suggested that an imbalance between the direct and indirect pathway of the basal ganglia plays a role in how the brain lesion in dyskinetic CP effects movements (i.e. the direct pathway is responsible for the control of voluntary movement and the indirect pathway for the inhibition of involuntary movements) [4, 94]. Therefore the assessment methods of control of voluntary movements versus inhibition of involuntary movements or a combination thereof might help to gain more knowledge how brain abnormalities result in dystonia and choreoathetosis in dyskinetic CP.

Some articles of the current review could be classified within ICF-CY level of activities and participation. However, none of the methods actually assessed activities within the daily environment of the participants. Therefore it is questionable whether results can be generalized to real-life situations. A large number of children and young adults with dyskinetic CP are dependent on wheeled mobility (manual or powered), instrumented assessment of quality and quantity of wheelchair use might be worthwhile. Assessing the performance of wheeled mobility (or the performance of different control devices for powered wheeled mobility) within a virtual environment as recently reported [68] is a very interesting option for this group.

In the recent years wearable sensor techniques have increasingly been used for detecting specific movements of interest, e.g. stereotypical movement patterns in epilepsies as well as for activity monitoring in neurological disorders [95] including the general population of CP [96, 97]. However, no study was found specifically for dyskinetic CP. Wearable sensors might offer opportunities in monitoring dyskinetic movements outside the laboratory setting. Considering that severity of abnormal movements varies over time and is exacerbated by external stimuli, such as stress, pain, and noise [4], measuring during a longer period of time in the daily environment of children and young adults with dyskinetic CP might result in more reliable measures.

## Conclusion

Although this current review shows the potential of several instrumented methods to be used as objective outcome measures in dyskinetic CP, their methodological quality is still unknown. Future development should focus on evaluating their clinimetrics, including validating against clinical meaningfulness. New technological developments should aim for measurements that can be applied outside the laboratory. This is especially important for the group of severely impaired children and young adults with dyskinetic CP.

## Supplementary information

**Additional file 1.** Search strategy.

**Additional file 2.** Overview of all included studies.

**Additional file 3.** Overview of studies that report correlation of parameters to a clinical comparator instrument.

**Additional file 4.** Overview of studies that report on comparison to a control group or distinguish between subgroups within the cerebral palsy (i.e. dyskinetic versus spastic).

**Additional file 5.** Overview of studies that report on reliability assessment.

**Additional file 6.** Overview of studies that report on pre/post intervention.

**Additional file 7:Table S3.** Characteristics of included studies (assessed body region, assessed ICF-CY categories, used instruments and technologies and measured parameters).

## Data Availability

All data generated or analyzed during this study are included in this published article and its supplementary information files.

## Abbreviations

BADS: Barry-Albright dystonia scale
BFMDRS: Burke-Fahn-Marsden dystonia rating scale
COPM: Canadian occupational performance measure
COSMIN: COnsensus-based Standards for the selection of health Measurement INstruments
CP: Cerbral palsy
CPCHILD: Child health index of life with disabilities
DIS: Dyskinesia impairment scale
GAS: Goal attainment scaling
GMFCS: Gross motor functioning classification system
HAT: Hypertonia assessment tool
ICC: Intraclass correlation coefficient
ICF-CY: International classification of functioning, disability and health for children and youth
MACS: Manual ability classification system
PEDI: Pediatric evaluation of disability inventory
PICOS: Participants, intervention, comparison, outcome, study design
PRISMA: Preferred reporting items for systematic reviews and meta-analyses
QUEST: Quality of upper extremity skills test
sEMG: surface electromyography
UDRS: Unified dystonia rating scale
