# Supplementary material for: Instrumented assessment of motor function in dyskinetic cerebral palsy: a systematic review
Source: J Neuroeng Rehabil. 2020 Mar 5;17:39. doi: 10.1186/s12984-020-00658-6 (PMC7057465; doi:10.1186/s12984-020-00658-6)
Supplement: Supplementary file 1 — Additional file 1. Search strategy. [file 12984_2020_658_MOESM1_ESM.pdf]

Additional file 1: Search strategy

Date of searches: **26-11-2019**

<u>Databases:</u>

PubMed	: 1504
Embase.com	: 2415
Scopus	: 3404
Totaal	: 7323
Additional records identified through other sorces:	: 4
Totaal na ontdubbelen	: 4537

<u>PubMed:</u>

#1 Cerebrale parese, dyskinesias & kinderen

("Cerebral Palsy"[Mesh] OR CP[tiab] OR "Cerebral Palsy"[tiab] OR "Cerebral Palsies"[tiab])

#2 Dyskinesias

("Dyskinesias"[Mesh:NoExp] OR "Athetosis"[Mesh] OR "Chorea"[Mesh:NoExp] OR "Dystonia"[Mesh:NoExp] OR dyskine*[tiab] OR diskine*[tiab] OR dyscine*[tiab] OR atheto*[tiab] OR dystonia[tiab] OR athetoid[tiab] OR "abnormal involuntary movement"[tiab])

#3 children 6-24 (child, adolescent, young adult)

"Young adult"[mesh] OR "Child"[mesh] OR "Minors"[mesh] OR "Adolescent"[mesh] OR child*[tw] OR adolescen*[tw] OR pediatric*[tw] OR paediatric*[tw] OR pube*[tw] OR juvenil*[tw] OR school*[tw] OR boy[tw] OR boys[tw] OR girl[tw] OR girls[tw] OR youth[tw] OR youths[tw] OR teen[tw] OR teens[tw] OR teenager*[tw] OR prepube*[tiab] OR preadolescen*[tiab] OR young*[tiab]

#4 instrumented measurements

"Biomechanical Phenomena"[Mesh:NoExp] OR "Biomedical Technology Assessment"[Mesh] OR "Accelerometry"[Mesh] OR "Ambulatory Monitoring"[Mesh] OR "Torque"[Mesh] OR "Kinetics"[Mesh] OR "Electromyography"[Mesh] OR "Video recording"[Mesh:NoExp] OR "Videotape Recording"[Mesh] OR "Computers, Handheld"[Mesh] OR torque*[tiab] OR biomechani*[tiab] OR Kinematic*[tiab] OR kinetic*[tiab] OR angle*[tiab] OR force*[tiab] OR motion[tiab] OR acceler*[tiab] OR rotation[tiab] OR velocity[tiab] OR velocities[tiab] OR speed[tiab] OR overflow[tiab] OR spatiotemporal[tiab] or temporal-spatial[tiab] OR electromyo*[tiab] OR EMG[tiab] OR 3D[tiab] OR three-dimensional[tiab] OR sensor[tiab] OR sensors[tiab] OR wearable[tiab] OR computer*[tiab] OR robot*[tiab] OR electrophysiolog*[tiab] OR video[tiab] OR videotap*[tiab] OR videorec*[tiab] OR camera[tiab] OR GPS[tiab] OR I-pad[tiab] OR iPad[tiab] OR gyroscope[tiab] OR signal*[tiab] OR smart phone*[tiab] OR smartphone*[tiab] OR mobile phone*[tiab]

((#1 AND #2) OR (#2 AND #3)) AND #4

EMBASE.com:

#1 Cerebrale parese, dyskinesias & kinderen

'cerebral palsy'/exp OR CP:ti,ab OR 'Cerebral Palsy':ti,ab OR 'Cerebral Palsies':ti,ab

#2 Dyskinesias

'dyskinesia'/de OR 'athetosis'/exp OR 'chorea'/de OR 'dystonia'/exp OR dyskine*:ti,ab OR diskine*:ti,ab OR dyscine*:ti,ab OR atheto*:ti,ab OR dystonia:ti,ab OR athetoid:ti,ab OR 'abnormal involuntary movement':ti,ab

#3 children 6-24 (child, adolescent, young adult)

'young adult'/exp OR 'child'/de OR 'school child'/exp OR 'boy'/exp OR 'girl'/exp OR 'adolescent'/exp OR child*:ti,ab OR adolescen*:ti,ab OR pediatric*:ti,ab OR paediatric*:ti,ab OR pube*:ti,ab OR juvenil*:ti,ab OR school*:ti,ab OR boy:ti,ab OR boys:ti,ab OR girl:ti,ab OR girls:ti,ab OR youth:ti,ab OR youths:ti,ab OR teen:ti,ab OR teens:ti,ab OR teenager*:ti,ab OR prepube*:ti,ab OR preadolescen*:ti,ab OR young*:ti,ab

#4 instrumented measurements

'biomechanics'/exp OR 'biomedical technology assessment'/exp OR 'accelerometry'/exp OR 'ambulatory monitoring'/exp OR 'torque'/exp OR 'kinetics'/de OR 'velocity'/exp OR 'electromyography'/exp OR 'videorecording'/exp OR 'personal digital assistant'/exp OR 'mobile phone'/exp OR 'wearable computer'/exp OR 'tablet computer'/exp OR torque*:ti,ab OR biomechani*:ti,ab OR Kinematic*:ti,ab OR kinetic*:ti,ab OR angle*:ti,ab OR force*:ti,ab OR motion:ti,ab OR acceler*:ti,ab OR rotation:ti,ab OR velocity:ti,ab OR velocities:ti,ab OR speed:ti,ab OR overflow:ti,ab OR spatiotemporal:ti,ab or 'temporal-spatial':ti,ab OR electromyo*:ti,ab OR EMG:ti,ab OR 3D:ti,ab OR 'three-dimensional':ti,ab OR sensor:ti,ab OR sensors:ti,ab OR wearable:ti,ab OR computer*:ti,ab OR robot*:ti,ab OR electrophysiolog*:ti,ab OR video:ti,ab OR videotap*:ti,ab OR videorec*:ti,ab OR camera:ti,ab OR GPS:ti,ab OR 'I-pad':ti,ab OR iPad:ti,ab OR gyroscope:ti,ab OR signal*:ti,ab OR 'smart phone*':ti,ab OR smartphone*:ti,ab OR 'mobile phone*':ti,ab OR iphone*:ti,ab

((#1 AND #2) OR (#2 AND #3)) AND #4

Scopus:

#1 Cerebrale parese, dyskinesias & kinderen

CP OR "Cerebral Palsy" OR "Cerebral Palsies"

#2 Dyskinesias

dyskine* OR diskine* OR dyscine* OR atheto* OR dystonia OR athetoid OR "abnormal involuntary movement"

#3 children 6-24 (child, adolescent, young adult)

child* OR adolescen* OR pediatric* OR paediatric* OR pube* OR juvenil* OR school* OR boy OR boys OR girl OR girls OR youth OR youths OR teen OR teens OR teenager* OR prepube* OR preadolescen* OR young*

#4 instrumented measurements

torque* OR biomechani* OR Kinematic* OR kinetic* OR angle* OR force* OR motion OR acceler* OR rotation OR velocity OR velocities OR speed OR overflow OR spatiotemporal or "temporal-spatial" OR electromyo* OR EMG OR 3D OR "three-dimensional" OR sensor OR sensors OR wearable OR computer* OR robot* OR electrophysiolog* OR video OR videotap* OR videorec* OR camera OR GPS OR "I-pad" OR iPad OR gyroscope OR signal* OR smartphone* OR "smart phone*" OR "mobile phone" OR iphone* (torque* OR biomechani* OR kinematic* OR kinetic* OR angle* OR force* OR motion OR acceler* OR rotation OR velocity OR velocities OR speed OR overflow OR spatiotemporal OR "temporal-spatial" OR electromyo* OR emg) OR (3d OR "three-dimensional" OR sensor OR sensors OR wearable OR computer* OR robot* OR electrophysiolog* OR video OR videotap* OR videorec* OR camera OR gps OR "i-pad" OR ipad OR gyroscope OR signal*) OR (smartphone* OR "smart phone*" OR "mobile phone" OR iphone*)
