# Supplementary material for: Instrumented assessment of motor function in dyskinetic cerebral palsy: a systematic review
Source: J Neuroeng Rehabil. 2020 Mar 5;17:39. doi: 10.1186/s12984-020-00658-6 (PMC7057465; doi:10.1186/s12984-020-00658-6)
Supplement: Supplementary file 2 — Additional file 2. Overview of all included studies. [file 12984_2020_658_MOESM2_ESM.pdf]

Additional file 2: Overview of studies

First author, year	Instruments / Technologies	Measured parameters	Number of participants		Dyskinetic cerebral palsy / Cerebral palsy with dystonia in patient group (%)	Age groups*	Short description or instruments / technologies
			Patients	Controls			
Androwis, 2015 (36)	-sEMG<br>-6DOF single position sensor<br>-Force sensor-	-Knee ROM<br>-Angular peak velocity<br>-Angular peak acceleration<br>-sEMG data	n=2	/	100%	-Adolescent<br>-Young adult	A 2Hz mechanical vestibular stimulation (15 minutes) is used. Participants are evaluated before and after stimulation to distinguish between dystonia and spasticity. Pendulum knee drop (PKD) test and mid-angle PKD test was administered before and after stimulation.
Beattie, 2016 (37)	-sEMG<br>-Video	-Mean number of active muscles during rest, quick stretch and volitional movement	n=13	n=8	46%	-Children<br>-Adolescents<br>-Young adults	sEMG was applied on four muscle groups of each leg. sEMG recordings and video were obtained during rest, quick stretch (in prone fast passive knee flexion, seated with feet and back supported: fast passive dorsiflexion) and volitional movement (active knee flexion in prone, active dorsiflexion in sitting, active knee extension in sitting, talking, playing a hand-held game in supported sitting).
Lebiedowska, 2004 (38)	-sEMG<br>-Electrogoniometer<br>-Force platforms<br>-3D motion capture system<br>-Force transducer	-Maximum voluntary isometric knee flexion and extension torques<br>-Co-contraction of antagonist during knee flexion and extension<br>-Knee tendon reflexes<br>-Resistance of knee joint during slow passive movement<br>-Torque of catch<br>-Kinematic gait parameters in the sagittal plane of knee range of motion<br>-Walking velocity	n=17	/	18%	-Children	A force transducer is used to assess resistance at the knee joint. Maximum voluntary isometric knee flexion and patella tendon reflexes are assessed. Electrogoniometer is used to measure knee flexion and extension. A 3D gait analysis is performed to evaluate kinematic gait parameters. Additional walking velocity is measured. Patients were classified into two groups (dystonia or spasticity).
Abel, 2003 (39)	-3D motion capture system	-Variability of ankle trajectory during swing	n=31	n=8	29%	-Children	The coefficient of variability is calculated from 3D trajectory of the left and right ankle markers (x,y,z trajectories)
Davids, 1999 (40)	-3D motion capture system	-Normalized dynamic base of support (step width)<br>-Step profile Step length/step width<br>-Total body maximal lateral acceleration	n=40	n=18	58%	-(Preschool) children<br>-Adolescents	3D motion analysis at self-selected speed to assess the presence of dyskinesia to develop a predictive model of dyskinesia based on gait parameters.
Petrarca, 2017 (41)	-3D motion capture system	-Kinematic data<br>-Movement pattern during gait and jumping	n=1	/	100%	-Child	Kinematics during gait cycle are compared to norm data. Also the timing of jumping was assessed.
Sangeux, 2016 (42)	-3D motion capture system	-Variablity of kinematic data – Overall Gait Variability Measure (OGVM)	n=416	/	2%	-(Preschool) children<br>-Adolescents	Measurements of the variability of gait kinematics were performed to provide an objective measure for dystonic gait disorders.
Berg, 1969 (43)	-sEMG<br>-Needle EMG	-Level of EMG activity during rest and activity<br>-Frequency spectrum	n=10	/	100%	-Adolescents<br>-Young adults	Muscle activity was measured during rest and during maximal isometric contraction after a fatigue protocol and following physical training for 20 minutes to evaluate voluntary control of muscles.

Additional file 2 (continued):

First author, year	Instruments / Technologies	Measured parameters	Number of participants		Dyskinetic cerebral palsy / Cerebral palsy with dystonia in patient group (%)	Age groups*	Short description or instruments / technologies
			Patients	Controls			
Kukke, 2011 (44)	-sEMG<br>-Jointed elbow brace belt-driven by a strong motor<br>-Optical position encoder	-Mean elbow extension velocity<br>-Peak elbow extension velocity<br>-Mean triceps EMG (overflow)<br>-Mean biceps EMG	n=11	n=11	100%	-Children<br>-Adolescents<br>-Young adults	Active elbow extension movements were assessed against each of four levels of resistance without perturbations or in each of two perturbation conditions (externally imposed extension stretch and stop during movement). Experiments were performed to assess the contribution of reflex activity, co-contraction and overflow in children with dyskinetic cerebral palsy.
Chu, 2009 (45)	-Force loadcell<br>-Biodex chair with arm device	-Force variability<br>-Signal dependence of noise (increase of variablity with force level)	n=11	n=11	100%	-Children<br>-Adolescents<br>-Young adults	The subjects were asked to produce a constant isometric elbow extension force, to evaluate force variability.
Van Doornik, 2009 (46)	-sEMG<br>-Force sensor<br>-Electrogoniometer<br>-Biodex chair<br>-Custom built apparatus to apply manual perturbations to elbow joint	-Correlation between elbow angle and sEMG activity<br>-sEMG activity during sinsuoidal perturbation<br>-Dependency of sEMG with velocity or position	n=8	/	100%	-Children<br>-Adolescents<br>-Young adults	sEMG was measured during passive flexion and extension movements with a constant amplitude (20°) with varying frequencies to measure reflex activity, position-dependent activation and velocity-dependent activation
Young, 2011 (47)	-Biodex chair<br>-Custom built apparatus to held shoulder abducted 90 degree and elbow flexed 90 degree<br>-sEMG<br>-Software to track a target by activating biceps and triceps	-Co-contraction (% of MVC)<br>-Tracking error	n=14	n=37	64%	-Adolescents	The effectiveness of biofeedback on muscle activity was measured. A tracking task was performed with an without visual feedback of co-contraction.
Niku, 1985 (48)	-Potentiometer<br>-Electrogoniometer<br>-Rigid and a flexible elbow brace<br>-Motion pattern generator, generating sinusoidal motions	-Movement frequency spectrum during:<br>*a visual tracking tests of sinusoidal motion<br>*a freewheeling test (move as fast as possible)	n=7	n=1	86%	-Adolescents (One subject 13 years others not stated)	A visual tracking task was performed regulated by flexion-extension movements of the arm as well as a 'free wheel test' (flexion extension movement) to assess involuntary and voluntary movement.
Chu, 2013 (49)	-Manipulandum rotating around a single axis (elbow rotation on a table, movement restricted to horizontal plane),<br>-Optical position encoder<br>-Software: showing movement of shuffelboard	-Mean velocity<br>-Intrinsic variabilty (Standard deviation of maximum velocity	n=16	n=16	100%	-Children<br>-Adolescents	The change of movement strategies was measured by changing the response of perceived motor variability in a simple virtual reality environment.

Additional file 2 (continued):

First author, year	Instruments / Technologies	Measured parameters	Number of participants		Dyskinetic cerebral palsy / Cerebral palsy with dystonia in patient group (%)	Age groups*	Short description or instruments / technologies
			Patients	Controls			
Chu, 2016 (50)	-Manipulandum rotating around a single axis (elbow rotation on a table, movement restricted to horizontal plane)<br>-Optical position encoder<br>-Software: showing movement of bal	-Performance error: distance error of virtual bal to target<br>-Reduction of performance error<br>-T- Cost (tolerance, costs with respect to optimal performance): Difference in mean error of location between the actual data and the ideal data<br>-Timing error<br>-Release time window	n=10	n=10	100%	-Children<br>-Adolescents	Throwing a virtual ball (control of position and velocity) was used to assess strategies to tolerate high movement variability (in dystonia);
Gordon, 2006 (51)	-sEMG<br>-Rigidtiy analyzer<br>-3D-motion capture system	-Total joint excursion (shoulder, elbow, wrist) of the resting arm (kinematic overflow)<br>Reaching performance:<br>-Peak velocity<br>-Wrist path ratio (curvature)<br>-End point error (overshoot or undershoot)<br>-Hold distance	n=13	n=8	8%	-Children<br>-Adolescents	Rigidity analyzer (i.e. a specialized pneumatic cuff with force sensor and gyroscope) was used to assess spasticity during passive movement with three velocities (slow, intermediate, fast); sEMG was used to measure reflex response; Dystonia and reaching performance were assessed using kinematic measures: Dystonia was quantified by measuring the amount of overflow movement in a resting arm that was caused by tapping the fingers of the contralateral arm. Reaching task: a 4-cm ball target: reach and touch the ball, hold
Butler, 2010 (52)	-3D motion capture system	-Movement time<br>-Movement time of Reach & Grasp cyle (i.e. reach forward and grasp, transport to mouth, transport back and release)<br>-Index of curvature during reach<br>-Peak velocity<br>-Number of movement units during the Reach & Gasp cycle (i.e. number of acceleration–decelerations in the velocity profile of the wrist marker)	n=12	n=25	58%	-(Preschool) children<br>-Adolescents	Temporal-spatial parameters during a reach & grasp cycle were measured to assess upper limb function.
Butler, 2012 (53)	-3D motion capture system	-Movement time<br>-Index of curvature during reach<br>-Number of movement units during the Reach & Gasp cycle (i.e. number of acceleration–decelerations in the velocity profile of the wrist marker)<br>-Angular velocity of elbow extension during reach<br>-Ratio of the peak velocity during 2 phases<br>-Peadiatric Upper Limb Motion Index (PULMI)	n=25	n=30	36%	-Children<br>-Adolescents	3D position data are recorded during a reach & grasp cycle and summarized as the Peadiatric Upper Limb Motion Index (PULMI) to quantify upper limb function in children with cerebral palsy.

Additional file 2 (continued):

First author, year	Instruments / Technologies	Measured parameters	Number of participants		Dyskinetic cerebral palsy / Cerebral palsy with dystonia in patient group (%)	Age groups*	Short description or instruments / technologies
			Patients	Controls			
Damiano, 2010 (54)	-3D motion capture system	-Reach velocity<br>-Path length<br>-Overflow to non-moving limb<br>-Ability to maintain a static arm posture	n=6	n=6	100%	-Children<br>-Adolescents	3D full-body kinematic analysis during functional movements (reach to target, self-selected and fast speed, shoulder flexion and abduction, and 5 seconds hold of the end posture of a flexion and abduction tasks
De Campos, 2014 (55)	-3D motion capture system	-Intralimb coordination: shoulder flexion/elbow extension correlation<br>-Reach time / Hold time<br>-Hand orientation error	n=8	n=9	100%	-Children<br>-Adolescents<br>-Young adults	A combination of sensory and motor assessments was performed using reach kinematics and tactile sensation.
Kukke, 2016 (56)	-3D motion capture system	-Hand aperture<br>-Movement time (between start of reach, hand-object contact time, object lift off)<br>-Atypical kinematics score (global score to summarize deviations from typical)	n=11	n=9	100%	-Children<br>-Adolescents<br>-Young adults	3D kinematic analysis of a reach to grasp task. Intra-limb coordination was studied using principal component analysis.
Elliott, 2011 (57)		-Movement time<br>-Directness index (ratio of actual path versus shortest path)<br>-Normalized jerk<br>-% time in primary movement<br>-% normalised jerk in primary movement	n=16	/	31%	-Children<br>-Adolescents	3D upper limb analysis was performed during four upper limb tasks (reach forward, reach forward to an elevated position, reach sideways to an elevated position and hand to mouth and down)
Sanger, 2006 (58)	-3D motion capture system	Measure of variability:<br>*Signal-to-noise ratio (ratio of first principal component of the joint velocity time serie to the sum of the remaining 10 components)<br>Measure of trajectory:<br>*Index of curvature<br>*Mean jerk during finger-to-nose task	n=7	n=21	100%	-(preschool) children<br>-Adolescents	During a 'finger-to-nose' task kinematics were measured to assess variability of movements.
Malfait, 2007 (59)	-3D motion capture system<br>-sEMG	- Average co-contraction over flexion/extension (finger-to-nose):<br>*Dot product of the EMG between biceps/triceps<br>*Minimum value of EMG between bicpes/triceps<br>-EMG activation levels during each phases<br>-Duration of whole movement circle<br>-Duration of phases:<br>*Acceleration/deceleration/Pause<br>-Maximum flexion/extension velocity<br>-Maximum elbow rotation<br>-Coefficient of variation of each measure	n=7	n=7	57%	-(preschool) children<br>-Adolescents	A 'finger-to-nose' tasks was applied to assess flexion-extension movement of the elbow concerning kinematics and muscle activation.

Additional file 2 (continued):

First author, year	Instruments / Technologies	Measured parameters	Number of participants		Dyskinetic cerebral palsy / Cerebral palsy with dystonia in patient group (%)	Age groups*	Short description or instruments / technologies
			Patients	Controls			
Pons, 2017 (60)	-3D motion capture system	-Index of dystonia (kinematic measure of overflow) during 'drinking' task<br>-Target accuracy (reach-and-point task):<br>*Hold distance<br>*End point error<br>During 5 functional upper extremity tasks and 4 reach-to-graps tasks:<br>*Movement duration<br>*Average and maximum linear velocity<br>*Index of curvature<br>*Joint angles	n=7	n=8	100%	-Children<br>-Adolescents	A movement protocol with 5 functional tasks and 4 reach to grasp tasks was performed, measured by 3D motion capture.
Kawamura, 2012 (61)	-3D motion capture system	-Kinematic dystonia measure (kinematic overflow: summation of joint angle movement of wrist, elbow and shoulder)	n=11	n=6	72%	-(preschool) children<br>-Adolescents	The amount of involuntary movement with 3D motion capture in a upper extremity triggered by tapping of the opposite hand or eye blinking.
Legros, 2004 (62)	-Three-axis piezoceramic accelerometer	-Integral/area under the curve of acceleration power spectrum during rest and posture	n=14	n=5	36%	-Children<br>-Adolescents<br>-Young adults	Accelerometric activity during rest (lying) and posture (arm at 45 degree) was quantified in the upper dominant limb. Data is used to quantify segmental activity of the dominant arm
Sanger, 2007 (63)	-Shape Tape	-Maximal velocity of outward reaching	n=7	/	100%	-(preschool) children<br>-Adolescents	Speed during forward reaching was measured to assess effects of botulinum toxin A. The task was adapted from finger-to-nose test
Liyanagamage, 2017 (64)	-sEMG	-Movement time<br>-Throughput (ratio of index of difficulty to movement time calculated by Fitts' Law)<br>-Muscle use (ratio of EMG in the vibrated muscle to non-vibrated muscle)	n=11	n=14	55%	-Children<br>-Adolescents<br>-Young adults	The effect of scaled vibratory feedback is used in a bimanual muscle-control task. Muscle activation and relaxation of the left and right biceps muscle (measured by sEMG) control the position and movement of a line on a computer screen.
Nwaobi, 1987 (65)	-sEMG<br>-Touch activiated switch	-Movement time<br>-EMG activity	n=1	/	100%	-Child	The effects of restraining the non-dominant arm on the function of the dominant arm were measured by. performance time and sEMG activity during activating a touch-activated switch.
Nwaobi, 1987 (66)	-Touch activiated switch	-Movement time	n=13	/	23%	-Children<br>-Adolescents	Participants were placed in different seating orientations and performed an abduction movement of the upper extremity (activating a touch-activated switch); performance time was measured; an electric goniometer was attached to the hip to monitor hip angle. During the test, the head and neck were supported in a collar and the trunk was supported by a backrest and a chest panel. Feet were placed on a platform (0 degrees DF, 90 degrees knee flexion)

Additional file 2 (continued):

First author, year	Instruments / Technologies	Measured parameters	Number of participants		Dyskinetic cerebral palsy / Cerebral palsy with dystonia in patient group (%)	Age groups*	Short description or instruments / technologies
			Patients	Controls			
Sanger, 2005 (67)	-Touch activated switch (three different sizes)	-Movement time<br>-Slope of regession lines of movement time on the logarithme of button width<br>-Slope of regression lines of speed on button width<br>-Slope and intercept of rgression lines of log variance on log mean speed	n=15	n=23	53%	-(preschool) children<br>-Adolescents	Different button size were used to assess relation between movement speed and button size
Rabhi, 2018 (68)	-Virtual environment simulating the wheelchair navigation	-Performance indices of user's path (path length, performance time, speed, number/duration of collisions, number of stops, succesfully passed targed points<br>-Performance indices from joystick (average amplitude, average speed)	n=3	/	67%	-Adolescents<br>-Young adults	A new control system for an electric wheelchair was tested in a virtual environment, assessing the path of the users as well as joystick data.
Bertucco, 2014 (69)	-Touchscreen tablet<br>-Custom made app – game to assess tradeoff between movement time and accuracy (Fitt's Law))	-Success rate<br>-Movement time<br>- Intercept and slope of linear regression of movement time as function of Indices of Difficulties (Fitt's law)<br>-Index of performance (1/slope of regression)<br>-Throughput (ratio of index of difficulty to movement time calculated by Fitts' Law)	n=16	n=15<br>n=13 (healthy adults)	44%	-Children<br>-Adolescents	Subjects were asked to touch target with different sizes on a touchscreen tablet with their index finger. A custom made program "Bubble burst" was used with two different instruction one that not touching the bubbles count or does not count (YES penalty, No penalty) The trade-off between time and accuracy was assess (Fitt's law)
Bertucco, 2015 (70)			n=16	n=15	44%		Same setup than Bertucco et al. 2014, without a penalty condition. The probability of an error was manipulated to assess the effect of the cost of error on Fitt's law.
Young, 2011 (71)	-sEMG<br>-Software tracking a target by isometrically activating of intrinsic muscles	-Tracking error<br>-Overflow (sEMG)	n=16	n=36	56%	-Children<br>-Adolescents<br>-Young adults	sEMG in two intrinsic hand muscles of both hands (four muscles in total) was measured while participants attempted to perform an isometric tracking task using one of the muscles, influence of visual feedback
Young, 2013 (72)			n=10	/	40%		
Young, 2014 (73)			n=14	/	71%		
Bhanpuri, 2015, (74)	-sEMG<br>-Software tracking a target by isometrically activating of intrinsic muscles	-Tracking error in step tracking task and continuous task<br>-Overflow (sEMG) error in step tracking task and continuous task	n=9	/	67%	-Children<br>-Adolescents<br>-Young adults	sEMG from a single first dorsal interosseous muscle controls the vertical position of a cursor on a display visible to the patient; Patient perform a step tracking and a continuous tracking task with 10% of the individual maximum voluntary contraction; sEMG is measured on first dorsal interosseous and abductor digiti minimi of both hands. Tracking error and overlow is assessed.
Lunardini, 2016 (75)	-3D motion capture system<br>-sEMG	-Accuracy error<br>-Speed<br>-Task correlation index (relative contribution of muscle activity correlated with 8-figure task)	n=2	/	100%	-Child<br>-Adolescent	A method to measure movement abnormalities during the performance of a continuous figure-eight writing task. Accuracy error, speed and task correlation index are evaluated.

Additional file 2 (continued):

First author, year	Instruments / Technologies	Measured parameters	Number of participants		Dyskinetic cerebral palsy / Cerebral palsy with dystonia in patient group (%)	Age groups*	Short description or instruments / technologies
			Patients	Controls			
Bertucco, 2019, (76)	-Touchscreen tablet<br>-sEMG	-Accuracy error<br>-Speed<br>-Ratio between error and speed<br>-Spatial variability<br>-Temporal variability<br>-Task correlation index (relative contribution of muscle activity correlated with 8-figure task)	n=7	n=7	43%	-Children<br>-Adolescents<br>-Young adults	The effect of vibro-tactile EMG-based feedback is assessed using a figure-eight writing task.
Choi, 2011 (77)	-Virtual handwriting training system, with PC connected to a haptic device that can provide force feedback	-Segmented movement time of transition and stroke-drawing<br>-Total movement time<br>-Time on-paper<br>-Time in-air<br>-Deviation of written trajectory from the ideal path	n=2	/	100%	-Children	Quantitative performance assessment of character writing before and after training with a haptic device providing visual and haptic cues.
Nicholson, 2001 (78)	-3D motion capture system	-Proximal stability at the trunk and distal stability at the wrist and hand<br>-Variation in movements between attempts of a particulair body part during reaching task in particular plane<br>-Smoothness trajectory of reach (visual comparision to normative data)	n=11	/	45%	-(preschool) children<br>-Adolescents	3D position data are recorded during a reach & grasp task
Garavaglia, 2017 (79)	-3D motion capture system	-Joint angles (thorax, shoulder, elbow, wrist)<br>Fingernail position	n=2	n=6	50%	-Adolescents	3D kinematic analysis during a pointing-forward task.
Cimolin, 2009 (80)	-3D motion capture system<br>-System for acquisition of pressure distribution placed on the seatback ond on headback of adaptive seating sytstem	-Trajectories of markers of head and trunk: Difference between initial position and end position after extensor thrust<br>-Initial position in anterior and vertical direction<br>-ROM of head, trunk and upper limb (difference between initial position and maximum valued during extensor thrust)<br>-Average jerk (smothness of movement) extensor thrust<br>-Peak of force on seatback and headpack during extensor thrust	n=10	/	100%	-Children	3D kinematics and pressure distribution during extensor thrusts induced by an external stimulus (noise) while sitting in a dynamic seating system were measured. A comparison of kinematics and pressure distribution were compared between dynamic and rigid seating system

Additional file 2 (continued):

First author, year	Instruments / Technologies	Measured parameters	Number of participants		Dyskinetic cerebral palsy / Cerebral palsy with dystonia in patient group (%)	Age groups*	Short description or instruments / technologies
			Patients	Controls			
Man, 2007 (81)	-Camera Mouse<br>-Head-mouse emulator switch<br>-Cross scanner (i.e. mouse-like pointer interface software) activiated by eye movement;<br>-Eye Gaze tracker<br>-Software: Point and-click test, based on the principle of Fitt's Law	-Movement time<br>-Accuracy rate	n=2	/	100%	-Adolescents	Comparison of four different computer-access solutions (i.e. on keyboard pointer) by a software that assess movement time and accuracy.
Raya, 2010 (82)	-Intertial sensor<br>-Software to control mouse pointer by inertial sensor	-Amplitude and frequency of movement<br>-Power spectral density versus time<br>-Standard deviations of angular head velocity	n=3	/	67%	-Children<br>-Adolescents	Capture of motion with inertial interface to measure involuntary movement by assessing angular velocity, acceleration and angular position of the head.
Saavedra, 2010 (83)	-3D motion capture system	-Head movement (sway parameters in sagittal and frontal plane)<br>-Head displacement (RMS) with eyes closed and open	n=15	n=26<br>n=11 (healthy adults)	13%	-Children<br>-Adolescents	Motion tracking of head movement during eyes open/eyes closed trial with the instruction to sit still during unsupported sit and different support options
Yamamoto, 2019 (84)	-2D motion capture system	-Head segment angle (side-bending)<br>-Head angular velocity	n=1	/	100%	-Adolescent	Motion tracking of head side bending during use of different computer input devices

Age groups: preschool children: 2-5 years; children: 6-12 years; adolescents: 13-18 years; young adult: 19-24 years

sEMG=surface electromyography; DOF=Degree of freedom; PKD=Pendulum knee drop; ROM=Range of Motion
