# Supplementary material for: Instrumented assessment of motor function in dyskinetic cerebral palsy: a systematic review
Source: J Neuroeng Rehabil. 2020 Mar 5;17:39. doi: 10.1186/s12984-020-00658-6 (PMC7057465; doi:10.1186/s12984-020-00658-6)
Supplement: Supplementary file 3 — Additional file 3. Overview of studies that report correlation of parameters to a clinical comparator instrument. [file 12984_2020_658_MOESM3_ESM.pdf]

Additional file 3: Overview of studies that report correlation of parameters to a clinical comparator instrument

	Measured parameters	*Comparator instrument*	*Correlation*	*p-value*
Abel,<br>2003 (39)	-Variability of ankle trajectory during swing	-Visual analog scale rating of swing variability	r=0.79	p<0.001
Young,<br>2011 (47)	-Co-contraction (% of MVC)	-Co-contraction correlation to BADS arm	r=0.68	p<0.001
Gordon,<br>2006 (51)	-Kinematic overflow<br>-Peak velocity<br>-Curvature of path<br>-Hold distance<br>-End point error	-Kinematic overflow correlation to BADS<br>-Peak velocity correlation to BADS<br>-Curvature of the path correlation to BADS<br>-Hold distance correlation to BADS<br>-End point error correlation to BADS	r=0.75<br>r=0.35<br>r=0.70<br>r=0.39<br>r=-0.65	p<0.005<br>p=0.24<br>p=0.008<br>p=0.19<br>p=0.02
Butler,<br>2010 (52)	-Movement time<br>-Index of curvature during reach<br>-Number of movement units during the reach, transport to mouth and total (i.e. number of acceleration–decelerations in the velocity profile of the wrist marker)	-Movement time correlation to MACS level<br>- Index of curvature correlation to MACS level<br>-Number of movement units during reach<br>to MACS level<br>-Number of movement units during transport<br>to mouth to MACS level<br>-Total number of movement units to MACS level	rho=0.96<br>rho=0.87<br><br>rho=0.91<br><br>rho=0.78,<br>rho=0.91,	p<0.001<br>p<0.001<br><br>p<0.001<br><br>p=0.003<br>p<0.001
Butler,<br>2012 (53)	-Peadiatric Upper Limb Motion Index (PULMI)	-Peadiatric Upper Limb Motion Index (PULMI) correlation to MACS level	rho=-0.78	p<0.0001<br>(in whole CP group)
De Campos,<br>2014 (55)	-Intralimb coordination: shoulder flexion/elbow extension correlation<br>-Reach time<br>-Hand orientation error<br>-Hold time	-Shoulder flexion/elbow extension correlation<br>to BFMDRS arm<br>-Reach time correlation to BFMDRS arm,<br>-Hand orientation error correlation to BFMDRS arm<br>-Hold time correlation to BFMDRS arm:	 rho=0.64<br>rho=0.81<br>rho= 0.45<br>rho=0.85;	 p=0.013<br>p<0.001<br>p=n.s.<br>p<0.001
Kukke,<br>2016 (56)	-Atypical kinematics score (global score to summarize deviations from typical movement)	-Atypical kinematic score correlation<br>to BFMDRS arm;	 rho=0.70	 p<0.0001
Sanger,<br>2006 (58)	-Signal-to-noise ratio (ratio of first principal component of the joint velocity time serie to the sum of the remaining 10 components)	-Signal-to-noise ratio correlation to BADS arm<br>to BFMDRS arm<br>to UDRS arm	 <br>r=0.82	p=n.s.<br>p=n.s.<br>p<0.004
Malfait,<br>2007 (59)	-Co-contraction<br>-Duration of pause phase	-Co-contraction correlation to BADS arm,<br>to BFMDRS arm<br>, to UDRS arm<br>-Duration of pause correlation to BADS<br>to BFMDRS<br>to UDRS	r=0.44<br>r=0.20<br>r=0.23<br>r=0.57<br>r=0.64<br>r=0.71	p=0.326<br>p=0.666<br>p=0.618<br>p=0.177<br>p=0.123<br>p=0.074
Pons,<br>2017 (60)	-Index of dystonia (kinematic measure of overflow)<br>-Target accuracy	-Index of dystonia correlation to BFMDRS arm<br>-Target accuracy correlation to BFMDRS arm	r=0.82	p=0.02<br>p=n.s.
Kawamura,<br>2012 (61)	-Kinematic dystonia measure (kinematic overflow: summation of joint angle movement of wrist, elbow and shoulder)	-Kinematic dystonia measure during hand tapping<br>correlation to BADS total:<br>to BADS arm<br>to QUEST<br>-Kinematic dystonia measure during eye blinking<br>correlation (after outlier removal) to BADS total:,<br>to BADS arm<br>to QUEST	 r=0.79<br>r=0.76<br>r= -0.60<br><br>r=0.41<br>r=0.72<br>r=-0.17	 p=0.003<br>p=0.007<br>p=0.05<br><br>p=0.24<br>p=0.02<br>p=0.63
Legros,<br>2004 (62)	-Integral/area under the curve of acceleration power spectrum during rest and posture	-Integral under the curve during rest correlation<br>to BFMDRS<br>to BFMDRS arm<br>-Integral under the cure during posture<br>to BFMDRS:<br>to BFMDRS arm	 rho=0.58	 p<0.01<br>p=n.s.<br><br>p=n.s.<br>p=n.s.
Bertucco,<br>2014 (69)	-Movement time<br>-Throughput (ratio of index of difficulty to movement time calculated by Fitts' Law)	-Movement time correlation to BADS arm<br>-Throughput correlation to BADS arm,	r =0.33<br>r=0.28	p<0.05<br>p<0.05
Young,<br>2011 (71)	-Tracking error<br>-Overflow (sEMG)	-Tracking error correlation to BADS arm<br>-Overflow (sEMG) correlation to BADS arm,	r=0.73<br>r=0.56	p<0.001<br>p<0.001
Young,<br>2013 (72)	-Tracking error<br>-Overflow (sEMG)	-Tracking error correlation to BADS arm,<br>-Overflow (sEMG) correlation to BADS arm,	r=0.28<br>r=0.28	p=not rep.<br>p=not rep.
Young,<br>2014 (73)	-Tracking error<br>-Overflow (sEMG)	-Tracking error correlation to BADS arm<br>-Overflow (sEMG) correlation to BADS arm,	r=0.59<br>r=0.50	p= not rep.<br>p= not rep..

BADS= Barry-Albright Dystonia scale ;BFMDRS = Burke-Fahn-Marsden Dystonia Rating Scale; MACS= Manual Ability Classification System ; QUEST=Quality of Upper Extremity Skills Test score; UDRS=Unified Dystonia Rating Scale; n.s.=not significant; not rep.=not reported
