# Supplementary material for: Instrumented assessment of motor function in dyskinetic cerebral palsy: a systematic review
Source: J Neuroeng Rehabil. 2020 Mar 5;17:39. doi: 10.1186/s12984-020-00658-6 (PMC7057465; doi:10.1186/s12984-020-00658-6)
Supplement: Supplementary file 4 — Additional file 4. Overview of studies that report on comparison to a control group or distinguish between subgroups within the cerebral palsy (i.e. dyskinetic versus spastic). [file 12984_2020_658_MOESM4_ESM.pdf]

Additional file 4 : Overview of studies that report on comparison to a control group or distinguish between subgroups within the cerebral palsy (i.e. dyskinetic versus spastic)

	Measured parameters	*Comparison between subgroups or to control group*	*Differences between groups*
Beattie, 2016 (37)	-Mean number of active muscles during rest, quick stretch and volitional movement	CP with dystonia (n=6)<br>Subjects with spasticity (n=7)<br>TD (n=8)	Groups differed on the mean number of active muscle during volitional movement (p=0.005) with the dystonic group showing the highest mean number (overflow from active motion) and with the spastic groups showing the highest mean number during quick stretch (p=0.017). Groups did not differ during rest.
Lebiedowska, 2004 (38)	-Maximum voluntary isometric knee flexion and extension torques<br>-Co-contraction of antagonist during knee flexion and extension<br>-Knee tendon reflexes<br>-Resistance of knee joint during slow passive movement<br>-Torque of catch<br>-Kinematic gait parameters in the sagittal plane of knee range of motion<br>-Walking velocity	CP with dystonia (n=3)<br>CP without dystonia (spastic) (n=14)	Maximum isometric flexion and extension torques were lower in the dystonic group comparted to spastic group (p<0.001) with a greater co-contraction of antagonists (p<0.05).<br>Knee tendon reflexes were higher in the spastic group compared to dystonic group (p<0.05).<br>Hamstrings and rectus femoris activity was higher in the dystonic group during passive knee extension and extension with slow velocities compared to spastic group (p<0.05)<br>At the beginning of the motion of slow passive flexion the resistance was higher in the dystonic group compared to spastic group.<br>In the spastic group the torque of catch was larger compared to the dystonic group (p<0.05)<br>Knee ROM was smaller in the stance phase of walking in the dystonic group compared to spastic group (p<0.001).<br>The velocity of self-paced walking was lower in the dystonic group compared to spastic group (p<0.001).
Abel, 2003 (39)	-Variability of ankle trajectory during swing	Dyskinetic CP (n=9)<br>Ataxic CP (n=10)<br>CP without athetosis and ataxia (n=12)<br>Healthy young adults/adults (n=8)	Groups differ on variability of ankle trajectory during swing (ANOVA, p<0.05). However only within CP the groups did not differ (p=0.1).
Davids, 1999 (40)	-Normalized dynamic base of support (step width)<br>-Step profile Step length/step width<br>-Total body maximal lateral acceleration	Dyskinetic CP (n=23)<br>Spastic CP (n=17)<br>TD (n=18)	Groups differed on all three parameters adequate (ANOVA, p=0.0001), with the dyskinetic group having greater dynamic base support and greater maximal lateral acceleration than the spastic and TD group. The dyskinetic had the smallest step profile followed by spastic CP, with TD having the highest value.
Sangeux, 2016 (42)	-Variablity of kinematic data – Overall Gait Variability Measure (OGVM)	CP with dystonia (n=8)<br>Spastic hemiplegic CP (n=134)<br>Spastic hemiplegic CP (n=240)<br>Spastic triplegic CP (n=24)	Group (CP subtype) was a significant factor in a general linear model with OGVM as the response. Post-hoc comparison showed no difference between spastic subtypes but the dystonic group had a significantly increased variability measured by OGVM.
Kukke, 2011 (44)	-Mean elbow extension velocity<br>-Peak elbow extension velocity<br>-Mean triceps EMG (overflow)<br>-Mean biceps EMG	CP with dystonia (n=11)<br>TD (n=11);	Slower mean and peak elbow extension velocity in dystonic group compared to control group (p<0.02). Increased muscle activity in the triceps and biceps in a subset of trial performed at similar movement velocity (p<0.0001)
Chu, 2009 (45)	-Force variability<br>-Signal dependence of noise (increase of variablity with force level)	Dyskinetic CP (n=10)/CP with dystonia (n=1)<br>TD (n=11)	Force variability was different between the groups (p<0.0001), with a higher variability in the dyskinetic/dystonic group. A weaker signal dependency of noise was shown in the dystonic group by comparing the regression value evaluating how much force variability was explained by target force between groups (p<0.0001)
Young, 2011 (47)	-Co-contraction (% of MVC)<br>-Tracking error	Primary (n=2) and secondary dystonia including CP (n=12)<br>TD (n=37)	Dystonic group had more co-contraction than control group (p=0.015) and a greater tracking error (p=0.001)
Niku, 1985 (48)	-Movement frequency spectrum during:<br>*a visual tracking tests of sinusoidal motion<br>*a freewheeling test (move as fast as possible)	Dyskinetic CP (n=5)<br>TD (n=1)	No statistical comparison. Differences in frequency spectrum observed during both tracking and freewheeling test.
Chu, 2013 (49)	-Mean velocity<br>-Intrinsic variabilty (Standard deviation of maximum velocity	CP with dystonia (n=16)<br>TD (n=16);	Both groups lowered their mean velocity when their displayed velocity was increased (p<0.001)<br>The intrinsic variability did not change with the change in displayed velocity in both groups.

Additional file 4 (continued):

	Measured parameters	*Comparison between subgroups or to control group*	*Differences between groups*
Chu, 2016 (50)	-Performance error: distance error of virtual bal to target<br>-Reduction of performance error<br>-T- Cost (tolerance, costs with respect to optimal performance): Difference in mean error of location between the actual data and the ideal data<br>-Timing error<br>-Release time window	CP with dystonia (n=10)<br>TD (n=10);<br>Healthy young adults/adults (n=10)	-Groups differed concerning performance error (ANOVA, factor group p=0.027)<br>-Reduction of performance error was similar between dyskinetic group and TD (p=0.471)<br>-T-cost was higher in dyskinetic group compared to TD (p=0.031)<br>-TD group had a better timing than dystonic group (p=0.001), timing error decreased in both groups (TD and dystonic) (p=0.005)<br>-Groups differed on release time window (p<0.001)-
Gordon, 2006 (51)	-Total joint excursion (shoulder, elbow, wrist) of the resting arm (kinematic overflow)<br>Reaching performance:<br>-Peak velocity<br>-Wrist path ratio (curvature)<br>-End point error (overshoot or undershoot)<br>-Hold distance	CP (n=13) with different severities of dystonia and spasticity (assessed by BADS and the Ashworth scale)<br>TD (n=8)	-Overflow between groups only described no statistical test<br>-Peak velocity was lower in CP group compared to TD (p=0.04)<br>-TD moved a straighter path (p=0.04)<br>-No significant difference in endpoint error (p=0.07) and hold distance (p=0.53)
Butler, 2012 (53)	-Movement time<br>-Index of curvature during reach<br>-Number of movement units during the Reach & Gasp cycle (i.e. number of acceleration–decelerations in the velocity profile of the wrist marker)<br>-Angular velocity of elbow extension during reach<br>-Ratio of the peak velocity during two phases<br>-Peadiatric Upper Limb Motion Index (PULMI)	Dyskinetic CP (n=9)<br>Spastic CP (n=12)<br>Mixed type CP (n=3)<br>TD (n=30)	-There were no differences in movement time, index of curvature during reach, angular velocity of elbow extension during reach and ratio of the peak velocity during two phases between the children with spastic and dyskinetic CP; however,<br>-Number of movement units was significantly greater among the children with dyskinetic versus spastic CP (p<0.04).<br>-PULMI scores were significantly lower among children with dyskinetic CP compared to spastic CP (p<0.02).
Damiano, 2010 (54)	-Reach velocity<br>-Path length<br>-Overflow to non-moving limb<br>-Ability to maintain a static arm posture	CP with hemidystonia (n=6)<br>TD (n=6)	-Reach velocity with affected arm was slower than in TD (p=0.03), path length did not differ<br>-Overflow and ability to maintain a static arm posture differ between groups (p<0.05)
De Campos, 2014 (55)	-Intralimb coordination: shoulder flexion/elbow extension correlation<br>-Reach time<br>-Hold time<br>-Hand orientation error	CP (due to unilateral perinatal stroke) with dystonia (n=7)<br>Spastic CP (n=17)<br>TD (n=18)	-Dystonic group had longer reach and hold times (p<0.01), and lower shoulder/elbow correlation (p<0.001) compared to the control group, while hand orientation error did not differ between groups<br>-Shoulder/elbow correlation, reach time, hold time, and hand orientation error on the dominant hand were not different between groups
Kukke, 2016 (56)	-Hand aperture<br>-Movement time (between start of reach, hand-object contact time, object lift off time);<br>-Atypical kinematics score (global score to summarize deviations from typical movement)	Hemiplegic CP with dystonia (n=11)<br>TD (n=9);	Movement times were longer in the dystonic group in the non-dominant hand compared to TD group (p<0.001)l and similar on the dominant hand (p=0.078).<br>Despite variability in hand aperture traces the median times of the maximum hand aperture was similar between groups for the dominant (p=0.201) and the non-dominant hand (p=0.503)<br>Hand aperture (during contract to rod) showed no group difference for the dominant hand (p=0.824) but for the non-dominant hand (p=0.024).<br>Groups differed on the atypical kinematics score on the non-dominant side (p<0.0001), but not on the dominant side
Elliott, 2011 (57)	-Movement time<br>-Directness index (ratio of actual path versus shortest path)<br>-Normalized jerk<br>-% time in primary movement<br>-% normalised jerk in primary movement	Dyskinetic CP (n=5)<br>Spastic CP (n=10)	Groups (at baseline) did differ on normalized jerk (p<0.001) with subjects from the dyskinetic group exhibit greater jerk, but not on %time in primary movement (p=0.788)

Additional file 4 (continued):

	Measured parameters	*Comparison between subgroups or to control group*	*Differences between groups*
Sanger, 2006 (58)	Measure of variability:<br>*Signal-to-noise ratio (ratio of first principal component of the joint velocity time serie to the sum of the remaining 10 components)<br>Measure of trajectory:<br>*Index of curvature<br>*Mean jerk during finger-to-nose task	Dyskinetic CP (n=7)<br>TD (n=21)	Mean jerk, signal-to-noise ratio and index of curvature differ with correction for age between the dyskinetic group and TD (p<0.05)
Malfait, 2007 (59)	- Average co-contraction over flexion/extension (finger-to-nose):<br>*Dot product of the EMG between biceps/triceps<br>*Minimum value of EMG between bicpes/triceps<br>-EMG activation levels during each phases<br>-Duration of whole movement circle<br>-Duration of phases:<br>*Acceleration/deceleration<br>*Pause<br>-Maximum flexion/extension velocity<br>-Maximum elbow rotation<br>-Coefficient of variation of each measure	Primary (n=3) and secondary dystonia due to CP (n=4)<br>TD (n=7)	Co-contraction of EMG calculated with two methods showed lower levels in the dystonic group compared to the control group (p=0.009, .0016)<br>Higher levels of EMG in TD during flexion deceleration than dystonic group (p>0.05)<br>Longer movement duration in dystonic group than control group (p=0.032), due to longer pauses at the target.<br>No significant difference in maximum elbow velocity during flexion/extension (p=0.106, 0.268))<br>Increased variability (coefficient of variation) for duration of the movement cycles (p>0.001) as well as for the maximum elbow velocities during the flexion phase (p=0.005). Variability of elbow velocity during extension phase did not differ between groups (p=0.123)
Pons, 2017 (60)	-Index of dystonia (kinematic measure of overflow) during 'drinking' task)<br>-Target accuracy (reach-and-point task):<br>*Hold distance<br>*End point error<br>During 5 functional upper extremity tasks and 4 reach-to-graps tasks:<br>*Movement duration<br>*Average and maximum linear velocity<br>*Index of curvature<br>*Joint angles	CP with dystonia (n=7)<br>TD (n=8)	Observed tendency to higher index of dystonia, longer movement durations, lower average velocities, increased index of curvature, shorter hold distance, larger end point error, greater wrist flexion, decreased elbow extension, increased trunk flexion-extension ROM in dystonic group compared to controls. No statistical analyses were performed.
Kawamura, 2012 (61)	-Kinematic dystonia measure (kinematic overflow: summation of joint angle movement of wrist, elbow and shoulder)	CP with dystonia (n=11)<br>TD (n=6)	Groups significantly differ on kinematic dystonia measures during the hand-tapping task (p=0.03) and the eye-blinking task (p=0.03)
Legros, 2004 (62)	-Integral/area under the curve of acceleration power spectrum during rest and posture	Primary (n=9) and secondary dystonia including CP (n=5)<br>TD (n=5);	Integral under the curve significantly differ between groups during rest (p<0.05) and posture (p<0.01) (before treatment)
Liyanagamage, 2017 (64)	-Movement time<br>-Throughput (ratio of index of difficulty to movement time calculated by Fitts' Law)<br>-Muscle use (ratio of EMG in the vibrated muscle to non-vibrated muscle)	Primary (n=3) and secondary dystonia including CP (n=8)<br>TD (n=14)	-Both groups followed patterns described by Fitt's law i.e. longer movement time with higher index of difficulties<br>-Movement time was higher in dystonic group than control group, and throughput was lower in the dystonic group (p<0.01)
Nwaobi, 1987 (66)	-Movement time	Dyskinetic CP (n=3)<br>Spastic CP (n=10)	Groups differences not statistically tested. Only descriptive

Additional file 4 (continued):

	Measured parameters	*Comparison between subgroups or to control group*	*Differences between groups*
Sanger, 2005 (67)	-Movement time<br>-Slope of regession lines of movement time on the logarithme of button width (Fitt's law)<br>-Slope of regression lines of speed on button width<br>-Slope and intercept of rgression lines of log variance on log mean speed	Primary (n=6) and secondary dystonia including CP (n=9)<br>TD (n=23);	-Movement time was longer in dystonic group compared to control group (p<0.0001).<br>-Steeper slope of regression lines of movement time on the logarithme of button width in dystonic group compared to control group (i.e. movement time decreases to greater amount when button size is larger in dystonic group) (p<0.05)<br>-Lower slope of regression line of speed on button width in dystonic group compared to control group (i.e. speed increases less in dystonic group with lager button size in dystonic group) (p<0.0001)<br>-For both groups the variance of speed increased with the average speed, but slopes differ between groups (p<0.0004) and intercept (p<0.006).
Bertucco, 2014 (69)<br>Bertucco, 2015 (70)	-Success rate<br>-Movement time<br>- Intercept and slope of linear regression of movement time as function of Indices of Difficulties (Fitt's law)<br>-Index of performance (1/slope of regression)<br>-Throughput (ratio of index of difficulty to movement time calculated by Fitts' Law)	Primary (n=6) and secondary dystonia including CP (n=10)<br>TD (n=15)<br>Healthy young adults/ adults (n=13)	-Success rate was lower in dystonic group compared to TD (p<0.05) or healthy adults (p=0.019)<br>-Movement time was slower for the dystonic group compared to TD (p<0.001) or adults (p<0.001)<br>-Higher intercept of the regression line of movement time on index difficulty in the dystonic group compared to TD (p<0.010) and healthy adults (p<0.001), and TD showed a higher intercept than healthy adults (p<0.001); the slope of the regression was steeper in dystonic group compared to healthy adults (p=0.013) but not when compared to TD.<br>-Index of performance was lower in dystonic group compared to TD (p<0.05) and healthy adults (p<0.006)<br>-Dystonic group had a lower throughput compared to TD (p=0.018 and adults (p<0.001))
Young, 2011 (71)	-Tracking error<br>-Overflow (EMG)	Primary (n=4) and secondary dystonia including CP (n=12)<br>TD (n=36)	-Dystonic group had more tracking error than the control group (p=0.001)<br>-Dystonic group had more overflow than the control group (p<0.008)
Bertucco, 2019, (76)	-Accuracy error<br>-Speed<br>-Ratio between error and speed<br>-Spatial variability<br>-Temporal variability<br>-Task correlation index (relative contribution of muscle activity correlated with 8-figure task)	Primary (n=2) and secondary dystonia including CP (n=7)<br>TD (n=7)	-Dystonic group had higher values of ratio between error and speed than the control group(p<0.05)<br>-Dystonic group had a higher spatial variability than the control group (p<0.05)<br>-Dystonic group had a lower task-correlation index (p<0.05)
Saavedra, 2010 (83)	-Head movement (sway parameters in sagittal and frontal plane)<br>-Head displacement (RMS) with eyes closed and open	Spastic CP (n=8)<br>Dyskinetic CP (n=2)	No statistical comparison between CP subgroups. An increased-RMS in dyskinetic group compared to spastic group when vision was present was observed

CP=cerebral palsy; TD= typically developing; BADS= Barry-Albright Dystonia scale; BFMDRS = Burke-Fahn-Marsden Dystonia Rating Scale; GMFCS=Gross Motor Classification System; HAT=Hypertonia Assessment Tool; MACS= Manual Ability Classification System ; QUEST=Quality of Upper Extremity Skills Test score; UDRS=Unified Dystonia Rating Scale;
CP=Cerebral palsy; TD= typically developing; sEMG=surface electromyography; MVC=maximum voluntary contraction; %=percentage; ROM= Range of Motion; RMS=Root Mean Square
