# Supplementary material for: Instrumented assessment of motor function in dyskinetic cerebral palsy: a systematic review
Source: J Neuroeng Rehabil. 2020 Mar 5;17:39. doi: 10.1186/s12984-020-00658-6 (PMC7057465; doi:10.1186/s12984-020-00658-6)
Supplement: Supplementary file 5 — Additional file 5. Overview of studies that report on reliability assessment. [file 12984_2020_658_MOESM5_ESM.pdf]

Additional file 5 : Overview of studies that report on reliability assessment

	Construct measured	*Type*	*ICCs/Correlation*	*SEM, SDC or LoA*
Butler, 2012 (53)	-Movement time<br>-Index of curvature during reach<br>-Number of movement units during the Reach & Gasp cycle<br>-Angular velocity of elbow extension<br>-Ratio of the peak velocity during two phases<br>-Peadiatric Upper Limb Motion Index (PULMI)	Intra-session reliability	Correlation all variables between two trials in dyskinetic CP rho=0.70-0.96, p≤0.001	Not reported
Kawamura, 2012 (61)	-Kinematic dystonia measure	Test-retest reliability (30 minutes later on the same day)	Hand-tapping task: ICC=0.95 (95% CI: 0.82, 0.99)<br>Eye blinking task: ICC=0.74 (95% CI:0.03, 0.93) (due to one outlier) after removal:<br>ICC=0.96 (95% CI:0.82, 0.99)	Hand-tapping task: LoA: ±80 degree<br>Eye blinking task:<br>±250 degree (due to one outlier)
Legros, 2004 (62)	-Area under the curve of acceleration power spectrum during rest and posture	Test-retest reliability	Correlation between first and second recording for rest and posture rho=0.97, p<0.05	Not reported
Nwaobi, 1987 (66)	-Movement time	Intra-session reliability<br>Test-retest reliability	Statistics not adequately reported	Not reported

ICCs= intraclass correlation coefficients; SEM= Standard error of measurement; SDC= smallest detectable difference; LoA= limits of agreement
