# Supplementary material for: Instrumented assessment of motor function in dyskinetic cerebral palsy: a systematic review
Source: J Neuroeng Rehabil. 2020 Mar 5;17:39. doi: 10.1186/s12984-020-00658-6 (PMC7057465; doi:10.1186/s12984-020-00658-6)
Supplement: Supplementary file 6 — Additional file 6. Overview of studies that report on pre/post intervention. [file 12984_2020_658_MOESM6_ESM.pdf]

Additional file 6 : Overview of studies that report on pre/post intervention

	Measured parameters	*Intervention*	*Comparison between subgroups (to control group) Or Comparator instrument*	*Result / Interpretation*
Berg, 1969 (43)	-Level of sEMG activity during rest and activity<br>-Frequency spectrum	Severe physical exercise		-No control group, no comparator instrument<br>-Observational description and comparison to previous measured TDs
Young, 2011 (47)	-Co-contraction (% of MVC)<br>-Tracking error	Visual feedback of biceps and triceps co-contraction	-primary dystonia (n=2) and secondary dystonia including CP (n=12)<br>-TD (n=36)	-Visual feedback reduced co-contraction in both groups (p<0.001) without increasing tracking error
Elliott, 2011 (57)	-Movement time<br>-Directness index (ratio of actual path versus shortest path)<br>-Normalized jerk<br>-% time in primary movement<br>-% normalised jerk in primary movement	Lycra® arm splints	Dyskinetic CP (n=5)<br>Spastic CP (n=10)	-% Time in primary movement increased in both groups (p=0.001 and p=0.048 respectively), while normalzied jerk decreased (p=0.001 and p=0.016 respectively)<br>-Whole group: Movement time, % time in primary movement, normalised jerk, % jerk in primary and-% jerk in secondary movement movement differed all between baseline and 3 month follow-up (p≤0.002). Directness index did not differ (p=0.410);
Legros, 2004 (62)	-Integral/area under the curve of acceleration power spectrum during rest and posture	Pallidal deep brain stimulation	-primary dystonia (n=9) and secondary dystonia (n=5) including subjects with CP<br>TD (n=5);	-Integral under the curve decreased during stimulation for rest and posture (p<0.01)<br>-No difference between patients and TD after treatment for integral under curve during rest and posture (p>0.05)
Sanger, 2007 (63)	-Maximal velocity of outward reaching	Botulinum Toxin Type B	CP with dystonia (n=7); Comparison to difference between two baseline measurements without treatment	-Change score of maximal velocity of outwards reaching between double baseline was different to the change score between baseline and the follow-up visits (p<0.05)
Liyanagamage, 2017 (64)	-Movement time<br>-Throughput (ratio of index of difficulty to movement time calculated by Fitts' Law)<br>-Muscle use (ratio of EMG in the vibrated muscle to non-vibrated muscle)	Biofeedback with vibration scaled to directly or inversely proportional to muscle activity on 4 days	Primary (n=3) and secondary dystonia including CP (n=8)<br>TD (n=14)	-Effect of different kinds of vibration was different for the groups. Throughput and movement time did not change due to vibration in the dystonic groups, while TD moved faster during constant, random and reverse vibration (p<0.003) and throughput differed in the TD group between proportional and random vibration (p<0.01)<br>-Muscle use increased in the dystonic group with proportional and reverse-scaled vibration (p<0.05), while in the control group constant vibration led to increased muscle use (p<0.05)
Nwaobi, 1987 (66)	-Movement time	Different seating orientations	Dyskinetic CP (n=3)<br>Spastic CP (n=10)	-Observational difference due to seating orientation in both groups
Young, 2011 (71)	-Tracking error<br>-Overflow (sEMG)	sEMG based visual feedback	Primary (n=4) and secondary dystonia including CP (n=12)<br>TD (n=36)	-Visual feedback had no significant effect on tracking error in both groups (feedback p=0.911, interaction p=0.333<br>-Visual feedback had a significant effect on overflow, effect was similar for both groups (feedback p<0.001, no interaction p=0.966)
Young, 2013 (72)	-Tracking error<br>-Overflow (sEMG)	Transcranial direct current stimulation	Primary and secondary dystonia (n=11) including dyskinetic CP	-Tracking error did not change with transcranial stimulation (p=0.495)<br>-Overflow was not reduced after treatment (p=0.340).
Young, 2014 (73)	-Tracking error<br>-Overflow (sEMG)	Transcranial direct current stimulation	Primary and secondary dystonia (n=11) including dyskinetic CP	-Tracking error did not change with transcranial stimulation (p>0.05)<br>-Overflow reduced when the hand contralateral to the cathode performed the task (p<0.05).
Bhanpuri, 2015, (74)	-Tracking error in step tracking task and continuous task<br>-Overflow (sEMG) error in step tracking task and continuous task	Transcranial direct current stimulation	Cathodal stimulation (n=7)<br>Anodal stimulation (n=6)	Cathodal group:<br>-No significant effect on tracking error or overflow during step tracking task (p>0.05)<br>-Improvement of tracking error (p<0.05), but an increase in overflow (p<0.05) for the continuous tracking task<br>Anodal group:<br>-Worsening of tracking error (p<0.05), but an decrease in overflow (p<0.05) for the step tracking task<br>-Worsening of tracking error (p<0.05),in overflow (increase) (p<0.05) for the continuous tracking task

Additional file 6 (continued):

	Measured parameters	*Intervention*	*Comparison between subgroups (to control group) Or Comparator instrument*	*Result / Interpretation*
Lunardini, 2016 (75)	-Accuracy error<br>-Speed<br>-Task correlation index (relative contribution of muscle activity correlated with 8-figure task)	sEMG based vibro-tactile feedback	Dyskinetic CP (n=2)	-Observational differences: decrease in accuracy error, an increase in task correlation index
Bertucco, 2019, (76)	Accuracy error<br>-Speed<br>-Ratio between error and speed<br>-Spatial variability<br>-Temporal variability<br>-Task correlation index (relative contribution of muscle activity correlated with 8-figure task)	sEMG based vibro-tactile feedback	Primary (n=2) and secondary dystonia including CP (n=7)<br>TD (n=7)	-Ratio between error and speed increased in both groups with vibro-tactile biofeedback ($p<0.05$)<br>-Spatial variability increased in both groups with vibro-tactile feedback ($p<0.001$)<br>-Task correlation index decreased in both groups with vibro-tactile biofeedback ($p<0.05$)
Cimolin, 2009 (80)	-Trajectories of markers of head and trunk: Difference between initial position and end position after extensor thrust<br>-Initial position in anterior and vertical direction<br>-ROM of head, trunk and upper limb (difference between initial position and maximum valued during extensor thrust)<br>-Average jerk (smothness of movement) extensor thrust<br>-Peak of force on seatback and headpack during extensor thrust	Rigid vs. dynamic seating system		-No significant difference in head trajectory between two seating systems ($p>0.05$)<br>-Trunk trajectory show larger and negative movements of the trunk in vertical directions in the rigid system ($p<0.05$)<br>-ROM were higher for head and trunk in the vertical direction compared to rigid ($p<0.05$).<br>-ROM of upper limb were lower in the dynamic seating system compared to rigid ($p<0.05$).<br>-Average jerk was lower in the dynamic seating system compared to rigid ($p<0.05$)<br>-Lower peak forces in the dynamic seating system compared to rigid ($p<0.05$)

CP=cerebral palsy; TD=Typically developing, ; sEMG=surface electromyography; MVC=maximum voluntary contraction; %=percentage; ROM= Range of Motion
