# Supplementary material for: Instrumented assessment of motor function in dyskinetic cerebral palsy: a systematic review
Source: J Neuroeng Rehabil. 2020 Mar 5;17:39. doi: 10.1186/s12984-020-00658-6 (PMC7057465; doi:10.1186/s12984-020-00658-6)
Supplement: Supplementary file 7 — Additional file 7:Table S3. Characteristics of included studies (assessed body region, assessed ICF-CY categories, used instruments and technologies and measured parameters). [file 12984_2020_658_MOESM7_ESM.pdf]

Additional file 7: Table S3: Characteristics of included studies (assessed body region, assessed ICF-CY categories, used instruments and technologies and measured parameters)

First author, year	Assessed body region	Assessed ICF-CY categories	Instruments / Technologies	Measured parameters	GMFCS amb. / non amb.	MACS	Comparison		Pre/post inter-vention	Reliability
							between groups	with clinical measure		
Androwis, 2015 (36)	Lower extremity	-Muscle tone / Motor reflex function	-sEMG<br>-6DOF single position sensor<br>-Force sensor-	-Knee ROM<br>-Angular peak velocity<br>-Angular peak acceleration<br>-EMG data	✗	✗	✗	✗	✗	✗
Beattie, 2016 (37)	Lower extremity	-Muscle tone / Motor reflex function	-sEMG<br>-Video	-Mean number of active muscles during rest, quick stretch and volitional movement	I-III	✗	✓	✗	✗	✗
Lebiedowska, 2004 (38)	Lower extremity	-Muscle power function<br>-Muscle tone / Motor reflex function<br>-Gait pattern function<br>-Walking	-sEMG<br>-Electrogoniometer<br>-Force platforms<br>-3D motion capture system<br>-Force transducer	-Maximum voluntary isometric knee flexion and extension torques<br>-Co-contraction of antagonist during knee flexion and extension<br>-Knee tendon reflexes<br>-Resistance of knee joint during slow passive movement<br>-Torque of catch<br>-Kinematic gait parameters in the sagittal plane of knee range of motion<br>-Walking velocity	amb.	✗	✓	✗	✗	✗
Abel, 2003 (39)	Lower extremity	-Gait pattern	-3D motion capture system	-Variability of ankle trajectory during swing	amb.	✗	✓	✓	✗	✗
Davids, 1999 (40)	Lower extremity	-Gait pattern	-3D motion capture system	-Normalized dynamic base of support (step width)<br>-Step profile Step length/step width<br>-Total body maximal lateral acceleration	amb.	✗	✓	✗	✗	✗
Petrarca, 2017 (41)	Lower extremity	-Gait pattern	-3D motion capture system	-Kinematic data<br>-Movement pattern during gait and jumping	amb.	✗	✗	✗	✗	✗
Sangeux, 2016 (42)	Lower extremity	-Gait pattern	-3D motion capture system	-Variablity of kinematic data – Overall Gait Variability Measure (OGVM)	I-III	✗	✓	✗	✗	✗
Berg, 1969 (43)	Lower extremity<br>Upper extremity	-Control of voluntary movement function	-sEMG<br>-Needle EMG	-Level of EMG activity during rest and activity<br>-Frequency spectrum	amb.& non amb	✗	✗	✗	✓	✗
Kukke, 2011 (44)	Upper extremity	-Muscle tone / Motor reflex function<br>-Control of voluntary movement function<br>-Involuntary movement function	-sEMG<br>-Jointed elbow brace belt-driven by a strong motor<br>-Optical position encoder	-Mean elbow extension velocity<br>-Peak elbow extension velocity<br>-Mean triceps sEMG (overflow)<br>-Mean biceps sEMG	✗	✗	✓	✗	✗	✗

Table 3 (continued):

First author, year	Assessed body region	Assessed ICF-CY categories	Instruments / Technologies	Measured parameters	GMFCS amb. / non amb.	MACS	Comparison between groups	Comparison with clinical measure	Pre/post inter-vention	Reliability
Chu, 2009 (45)	Upper extremity	-Muscle power function	-Force loadcell<br>-Biodex chair with arm device	-Force variability<br>-Signal dependence of noise (increase of variablity with force level)	✗	✗	✓	✗	✗	✗
Van Doornik, 2009 (46)	Upper extremity	-Muscle tone / Motor reflex function	-sEMG<br>-Force sensor<br>-Electrogoniometer<br>-Biodex chair<br>-Custom built apparatus to apply manual perturbations to elbow joint	-Correlation between elbow angle and sEMG activity<br>-sEMG activity during sinsuoidal perturbation<br>-Dependency of EMG with velocity or position	✗	✗	✗	✗	✗	✗
Young, 2011 (47)	Upper extremity	-Control of voluntary movement function	-Biodex chair<br>-Custom built apparatus to held shoulder abducted 90 degree and elbow flexed 90 degree<br>-sEMG<br>-Software to track a target by activating biceps and triceps	-Co-contraction (% of MVC)<br>-Tracking error	✗	✗	✓	✓	✓	✗
Niku, 1985 (48)	Upper extremity	-Control of voluntary movement function<br>-Involuntary movement function	-Potentiometer<br>-Electrogoniometer<br>-Rigid and a flexible elbow brace<br>-Motion pattern generator, generating sinusoidal motions	-Movement frequency spectrum during:<br>*a visual tracking tests of sinusoidal motion<br>*a freewheeling test (move as fast as possible)	✗	✗	✓	✗	✗	✗
Chu, 2013 (49)	Upper extremity	-Control of voluntary movement function	-Manipulandum rotating around a single axis (elbow rotation on a table, movement restricted to horizontal plane),<br>-Optical position encoder<br>-Software: showing movement of shuffelboard	-Mean velocity<br>-Intrinsic variabilty (Standard deviation of maximum velocity	✗	✗	✓	✗	✗	✗
Chu, 2016 (50)	Upper extremity	- Control of voluntary movement function	-Manipulandum rotating around a single axis (elbow rotation on a table, movement restricted to horizontal plane)<br>-Optical position encoder<br>-Software: showing movement of bal	-Performance error: distance error of virtual bal to target<br>-Reduction of performance error<br>-T- Cost (tolerance, costs with respect to optimal performance): Difference in mean error of location between the actual data and the ideal data<br>-Timing error<br>-Release time window	✗	✗	✓	✗	✗	✗

Table 3 (continued):

First author, year	Assessed body region	Assessed ICF-CY categories	Instruments / Technologies	Measured parameters	GMFCS amb. / non amb.	MACS	Comparison between groups	Comparison with clinical measure	Pre/post inter-vention	Reliability
Gordon, 2006 (51)	Upper extremity	-Muscle tone / Motor reflex function<br>-Control of voluntary movement function<br>-Involuntary movement function<br>-Hand and arm use	-sEMG<br>-Rigidtiy analyzer<br>-3D-motion capture system	-Total joint excursion (shoulder, elbow, wrist) of the resting arm (kinematic overflow)<br>Reaching performance:<br>-Peak velocity<br>-Wrist path ratio (curvature)<br>-End point error (overshoot or undershoot)<br>-Hold distance	✗	✗	✓	✓	✗	✗
Butler, 2010 (52)	Upper extremity	-Control of voluntary movement function<br>-Lifting and carrying objects	-3D motion capture system	-Movement time<br>-Movement time of different phases of Reach & Grasp cyle (i.e. reach forward and grasp, transport to mouth, transport back and release)<br>-Index of curvature during reach<br>-Peak velocity<br>-Number of movement units during the Reach & Gasp cycle (i.e. number of acceleration–decelerations in the velocity profile of the wrist marker)	✗	I-III	✓	✓	✗	✗
Butler, 2012 (53)	Upper extremity	-Control of voluntary movement function<br>-Lifting and carrying objects	-3D motion capture system	-Movement time<br>-Index of curvature during reach<br>-Number of movement units during the Reach & Gasp cycle (i.e. number of acceleration–decelerations in the velocity profile of the wrist marker)<br>-Angular velocity of elbow extension during reach<br>-Ratio of the peak velocity during two phases<br>-Peadiatric Upper Limb Motion Index (PULMI)	✗	I-III	✓	✓	✗	✓
Damiano, 2010 (54)	Upper extremity	-Control of voluntary movement function<br>-Involuntary movement function<br>-Hand and arm use	-3D motion capture system	-Reach velocity<br>-Path length<br>-Overflow to non-moving limb<br>-Ability to maintain a static arm posture	✗	II-IV	✓	✗	✗	✗

Table 3 (continued):

First author, year	Assessed body region	Assessed ICF-CY categories	Instruments / Technologies	Measured parameters	GMFCS amb. / non amb.	MACS	Comparison		Pre/post inter-vention	Reliability
							between groups	with clinical measure		
De Campos, 2014 (55)	Upper extremity	-Control of voluntary movement function<br>-Lifting and carrying objects	-3D motion capture system	-Intralimb coordination: shoulder flexion/elbow extension correlation<br>-Reach time<br>-Hold time<br>-Hand orientation error	✗	I-III	✓	✓	✗	✗
Kukke, 2016 (56)				-Hand aperture<br>-Movement time (between start of reach, hand-object contact time, object lift off time);<br>-Atypical kinematics score (global score to summarize deviations from typical movement)	✗	I-III	✓	✓	✗	✗
Elliott, 2011 (57)	Upper extremity	-Control of voluntary movement function<br>-Hand and arm use	-3D motion capture system	-Movement time<br>-Directness index (ratio of actual path versus shortest path)<br>-Normalized jerk<br>-% time in primary movement<br>-% normalised jerk in primary movement	✗	✗	✓	✗	✓	✗
Sanger, 2006 (58)	Upper extremity	-Control of voluntary movement function<br>-Hand and arm use	-3D motion capture system	Measure of variability:<br>*Signal-to-noise ratio (ratio of first principal component of the joint velocity time serie to the sum of the remaining 10 components)<br>Measure of trajectory:<br>*Index of curvature<br>*Mean jerk during finger-to-nose task	✗	✗	✓	✓	✗	✗
Malfait, 2007 (59)	Upper extremity	-Control of voluntary movement function<br>-Hand and arm use	-3D motion capture system<br>-sEMG	- Average co-contraction over flexion/extension (finger-to-nose):<br>*Dot product of the sEMG between biceps/triceps<br>*Minimum value of sEMG between bicpes/triceps<br>-EMG activation levels during each phases<br>-Duration of whole movement circle<br>-Duration of phases:<br>*Acceleration/deceleration<br>*Pause<br>-Maximum flexion/extension velocity<br>-Maximum elbow rotation<br>-Coefficient of variation of each measure	II-V	✗	✓	✓	✗	✗

Table 3 (continued):

First author, year	Assessed body region	Assessed ICF-CY categories	Instruments / Technologies	Measured parameters	GMFCS amb. / non amb.	MACS	Comparison		Pre/post inter-vention	Reliability
							between groups	with clinical measure		
Pons, 2017 (60)	Upper extremity	-Control of voluntary movement function<br>-Involuntary movement function<br>-Hand an arm use	-3D motion capture system	-Index of dystonia (kinematic measure of overflow) during ‘drinking’ task)<br>-Target accuracy (reach-and-point task):<br>*Hold distance<br>*End point error<br>During 5 functional upper extremity tasks and 4 reach-to-graps tasks:<br>*Movement duration<br>*Average and maximum linear velocity<br>*Index of curvature<br>*Joint angles	I-IV	I-III	✓	✓	✗	✗
Kawamura, 2012 (61)	Upper extremity	-Involuntary movement function	-3D motion capture system	-Kinematic dystonia measure (kinematic overflow: summation of joint angle movement of wrist, elbow and shoulder)	I-V	I-IV	✓	✓	✗	✓
Legros, 2004 (62)	Upper extremity	-Involuntary movement function	-Three-axis piezoceramic accelerometer	-Integral/area under the curve of acceleration power spectrum during rest and posture	✗	✗	✓	✓	✓	✓
Sanger, 2007 (63)	Upper extremity	-Control of voluntary movement function<br>-Hand and arm use	-Shape Tape	-Maximal velocity of outward reaching	✗	✗	✗	✗	✓	✗
Liyanagamage, 2017 (64)	Upper extremity	-Control of voluntary movement function	-sEMG	-Movement time<br>-Throughput (ratio of index of difficulty to movement time calculated by Fitts' Law)<br>-Muscle use (ratio of EMG in the vibrated muscle to non-vibrated muscle)	✗	✗	✓	✗	✓	✗
Nwaobi, 1987 (65)<br>Nwaobi, 1987 (66)	Upper extremity	-Control of voluntary movement function<br>-Hand and arm use	-sEMG<br>-Touch activiated switch	-Movement time<br>-sEMG activity	✗	✗	✗	✗	✓	✓
Sanger, 2005 (67)	Upper extremity	-Control of voluntary movement function<br>-Hand and arm use	-Touch activated switch (three different sizes)	-Movement time<br>-Slope of regession lines of movement time on the logarithme of button width (Fitt's law)<br>-Slope of regression lines of speed on button width<br>-Slope and intercept of rgression lines of log variance on log mean speed	✗	✗	✓	✗	✗	✗

Table 3 (continued):

First author, year	Assessed body region	Assessed ICF-CY categories	Instruments / Technologies	Measured parameters	GMFCS amb. / non amb.	MACS	Comparison between groups	Comparison with clinical measure	Pre/post inter-vention	Reliability
Rabhi, 2018 (68)	Upper extremity	-Control of voluntary movement function<br>-Moving around using equipment	-Virtual environment simulating the wheelchair navigation	-Performance indices of user's path (path length, performance time, speed, number/duration of collisions, number of stops, succesfully passed targed points<br>-Performance indices from joystick (average amplitude, average speed)	V	✘	✘	✘	✘	✘
Bertucco, 2014 (69)<br>Bertucco, 2015 (70)	Upper extremity / fingers	-Control of voluntary movement function<br>-Fine hand use	-Touchscreen tablet<br>-Custom made app – game to assess tradeoff between movement time and accuracy (Fitt's Law))	-Success rate<br>-Movement time<br>- Intercept and slope of linear regression of movement time as function of Indices of Difficulties (Fitt's law)<br>-Index of performance (1/slope of regression)<br>-Throughput (ratio of index of difficulty to movement time calculated by Fitts' Law)	✘	✘	✓	✓	✘	✘
Young, 2011 (71)<br>Young, 2013 (72)<br>Young, 2014 (73)	Upper extremity / fingers	-Control of voluntary movement function<br>-Involuntary movement function	-sEMG<br>-Software tracking a target by isometrically activating of intrinsic muscles	-Tracking error<br>-Overflow (EMG)	✘	✘	✓	✓	✓	✘
Bhanpuri, 2015, (74)	Upper extremity / fingers	-Control of voluntary movement function<br>-Involuntary movement function	-sEMG<br>-Software tracking a target by isometrically activating of intrinsic muscles	-Tracking error in step tracking task and continuous task<br>-Overflow (sEMG) error in step tracking task and continuous task	✘	✘	✘	✘	✓	✘
Lunardini, 2016 (75)	Upper extremity /fingers	-Control of voluntary movement function<br>-Fine hand use	-3D motion capture system<br>-sEMG	-Accuracy error<br>-Speed<br>-Task correlation index (relative contribution of muscle activity correlated with 8-figure task)	✘	✘	✘	✘	✓	✘
Bertucco, 2019, (76)	Upper extremity /fingers	-Control of voluntary movement function<br>-Fine hand use	-Touchscreen tablet<br>-sEMG	-Accuracy error<br>-Speed<br>-Ratio between error and speed<br>-Spatial variability<br>-Temporal variability<br>-Task correlation index (relative contribution of muscle activity correlated with 8-figure task)	✘	✘	✓	✘	✓	✘

Table 3 (continued):

First author, year	Assessed body region	Assessed ICF-CY categories	Instruments / Technologies	Measured parameters	GMFCS amb. / non amb.	MACS	Comparison between groups	Comparison with clinical measure	Pre/post inter-vention	Reliability
Choi, 2011 (77)	Upper extremity /fingers	-Control of voluntary movement function<br>-Fine hand use	-Virtual handwriting training system, with PC connected to a haptic device that can provide force feedback	-Segmented movement time of transition and stroke-drawing<br>-Total movement time<br>-Time on-paper/Time in-air<br>-Deviation of written trajectory from the ideal path	✘	✘	✘	✘	✘	✘
Nicholson, 2001 (78)	Trunk<br>Upper extremity	-Control of voluntary movement function<br>-Hand and arm use	-3D motion capture system	-Proximal stability at the trunk and distal stability at the wrist and hand<br>-Variation in movements between attempts of a particulair body part during reaching task in particular plane<br>-Smoothness trajectory of reach (visual comparision to normative data)	✘	✘	✘	✘	✘	✘
Garavaglia, 2017 (79)	Trunk<br>Upper extremity	-Control of voluntary movement function<br>-Hand and arm use<br>-Maintaining a body position	-3D motion capture system	-Joint angles (thorax, shoulder, elbow, wrist)<br>Fingernail position	✘	✘	✘	✘	✘	✘
Cimolin, 2009 (80)	Trunk<br>Head<br>Upper extremity	-Involuntary movement function<br>-Maintaining a body position	-3D motion capture system<br>-System for acquisition of pressure distribution placed on the seatback ond on headback of adaptive seating sytstem	-Trajectories of markers of head and trunk: Difference between initial position and end position after extensor thrust<br>-Initial position in anterior and vertical direction<br>-ROM of head, trunk and upper limb (difference between initial position and maximum valued during extensor thrust)<br>-Average jerk (smothness of movement) extensor thrust<br>-Peak of force on seatback and headpack during extensor thrust	V	✘	✘	✘	✓	✘
Man, 2007 (81)	Head	- Control of voluntary movement function	-Camera Mouse<br>-Head-mouse emulator switch<br>-Cross scanner (i.e. mouse-like pointer interface software) activiated by eye movement;<br>-Eye Gaze tracker<br>-Software: Point and-click test, based on the principle of Fitt's Law	-Movement time<br>-Accuracy rate	✘	✘	✘	✘	✘	✘

Table 3 (continued):

First author, year	Assessed body region	Assessed ICF-CY categories	Instruments / Technologies	Measured parameters	GMFCS amb. / non amb.	MACS	Comparison		Pre/post inter-vention	Reliability
Raya, 2010 (82)	Head	-Involuntary movement function	-Intertial sensor<br>-Software to control mouse pointer by inertial sensor	-Amplitude and frequency of movement<br>-Power spectral density versus time<br>-Standard deviations of angular head velocity	V	✘	✘	✘	✘	✘
Saavedra, 2010 (83)	Head	-Involuntary movement function<br>-Maintaining a body position	-3D motion capture system	-Head movement (sway parameters in sagittal and frontal plane)<br>-Head displacement (RMS) with eyes closed and open	I-III	✘	✓	✘	✘	✘
Yamamoto, 2019 (84)	Head	-Involuntary movement function<br>-Maintaining a body position	-2D motion capture system	-Head segment angle (side-bending)<br>-Head angular velocity	V	V	✘	✘	✘	✘

sEMG=surface electromyography; DOF=Degree of freedom; ROM=Range of Motion; OGVM= Overall Gait Variability Measure; MVC=maximum voluntary contraction; PULMI)=Peadiatric Upper Limb Motion Index ; %=percentage; RMS=Root mean amb=ambulatory; non amb.=non ambulatory; GMFCS=Gross motor functioning classification system; MACS=Manual Ability Classification System

✘ = information not available; ✓ = information available;
